# A role for HOX13 proteins in the regulatory switch between TADs at the *HoxD* locus

**DOI:** 10.1101/gad.281055.116

**Published:** 2016-05-15

**Authors:** Leonardo Beccari, Nayuta Yakushiji-Kaminatsui, Joost M. Woltering, Anamaria Necsulea, Nicolas Lonfat, Eddie Rodríguez-Carballo, Benedicte Mascrez, Shiori Yamamoto, Atsushi Kuroiwa, Denis Duboule

**Affiliations:** 1 Department of Genetics and Evolution, University of Geneva, 1211 Geneva 4, Switzerland;; 2 School of Life Sciences, Federal Institute of Technology, Lausanne, 1015 Lausanne, Switzerland;; 3 Division of Biological Science, Graduate School of Science, Nagoya University, Chikusa-ku, Nagoya 464-8602, Japan

**Keywords:** topologically associating domains (TADs), vertebrate limbs, regulatory landscapes, wrist, ankle, polycomb

## Abstract

During vertebrate limb development, *Hoxd* genes are regulated following a bimodal strategy involving two topologically associating domains (TADs) located on either side of the gene cluster. These regulatory landscapes alternatively control different subsets of *Hoxd* targets, first into the arm and subsequently into the digits. We studied the transition between these two global regulations, a switch that correlates with the positioning of the wrist, which articulates these two main limb segments. We show that the HOX13 proteins themselves help switch off the telomeric TAD, likely through a global repressive mechanism. At the same time, they directly interact with distal enhancers to sustain the activity of the centromeric TAD, thus explaining both the sequential and exclusive operating processes of these two regulatory domains. We propose a model in which the activation of *Hox13* gene expression in distal limb cells both interrupts the proximal *Hox* gene regulation and re-enforces the distal regulation. In the absence of HOX13 proteins, a proximal limb structure grows without any sign of wrist articulation, likely related to an ancestral fish-like condition.

## Abstract (precis)

During vertebrate limb development, *Hoxd* genes are regulated following a bimodal strategy involving two topologically associating domains (TADs) located on either side of the gene cluster. This work shows that the HOX13 proteins themselves help switch off the telomeric TAD. At the same time, they directly interact with distal enhancers to sustain the activity of the centromeric TAD, thus explaining both the sequential and exclusive operating processes of these two regulatory domains.

Vertebrate limbs have been a paradigm in our understanding of the emergence of patterns during evolution and development, in terms of both the molecules involved and the underlying principles (e.g., see Tabin and Wolpert 2007). Developing limb buds indeed implement all major signaling pathways as well as families of transcription factors known for their importance in the building of the embryo. This co-optation of key developmental functions to accompany the evolution of paired limbs was initially observed for the *HoxD* cluster (Dolle et al. 1989; Lewis and Martin 1989), which, together with the *HoxA* cluster (Haack and Gruss 1993; Yokouchi et al. 1995b), is critical for the formation of the proximal and distal pieces of our arms and legs (Davis et al. 1995; Fromental-Ramain et al. 1996; Kmita et al. 2005). While the exact function of HOX proteins during limb development remains to be fully assessed, they seem to be involved in the control of bone growth (Zákány and Duboule 1996; Villavicencio-Lorini et al. 2010; Gonzalez-Martin et al. 2014; Kuss et al. 2014) in both complementary and redundant manners (references in Zakany and Duboule 2007).

*Hoxa* and *Hoxd* genes are transcribed into precisely delimited domains within the incipient limb buds, which will determine the advent and positioning of future morphologies. For instance, *Hoxa13* determines the distal part of the growing limb, the digits, whereas its neighbor, *Hoxa11*, is a marker of the proximal limb piece, the forearm (Yokouchi et al. 1991; Nelson et al. 1996). The study of the regulations acting over the *HoxA* cluster to elicit these transcript patterns revealed the presence of long-range global enhancers (Lehoczky and Innis 2008) located within a flanking topologically associating domain (TAD) (Berlivet et al. 2013; Woltering et al. 2014); i.e., a chromatin structure where enhancer–promoter contacts as well as constitutive interactions are privileged (Dixon et al. 2012; Nora et al. 2012; Sexton and Cavalli 2015). In the case of *Hoxd* genes, genetic and molecular analyses have shown that their complex expression patterns are controlled by the successive implementation of global regulations contained within two flanking TADs, covering the neighboring gene deserts (Andrey et al. 2013).

In early limb buds, enhancers located within the telomeric TAD (T-DOM) control the transcription of a central group of genes, from *Hoxd8* to *Hoxd11*, into the presumptive forearm (Andrey et al. 2013). Soon after, in the most distal aspect of the limb bud, the T-DOM stops operating, and enhancers specific for digit expression, located within the centromeric TAD (C-DOM), become activated (Montavon et al. 2011). Therefore, the complete expression pattern of *Hoxd* genes involves a switch from a situation in which T-DOM is active and C-DOM is inactive to a state where C-DOM is active and T-DOM is inactive (see Lonfat and Duboule 2015). The existence of two separate and independent regulatory landscapes allows for the appearance of a stripe of nonexpressing cells between the two transcript domains. These cells express low levels of *Hox* genes and are thought to produce the mesopodial articulation; i.e., the wrist and ankle (Villavicencio-Lorini et al. 2010; Woltering and Duboule 2010). In this context, the switch in TAD regulations is key in the making and positioning of the mesopodium, an essential structure in the evolution of tetrapods, which allowed them to properly articulate their newly acquired digits.

The molecular mechanism controlling the switch from T-DOM to C-DOM at the *HoxD* locus is hard to study due to the high number of enhancers, their remote locations, and the scarcity of in vivo biological material. However, some evidence suggested that the HOX13 proteins themselves may have a negative impact on the transcription of forearm-specific *Hoxa* and *Hoxd* genes. First, the proximal boundary of the *Hoxa13* expression domain, which labels the distal end of the forearm, exactly matches the distal boundaries of the cellular domains expressing either *Hoxa11* or *Hoxd9*, *Hoxd10*, and *Hoxd11* (e.g., see Woltering and Duboule 2010). The separation between these expression domains is not observed in fish, where the *Hoxa13* and *Hoxa11* cellular territories overlap, suggesting an important evolutionary change (van der Hoeven et al. 1996; Metscher et al. 2005; Davis et al. 2007; Tamura et al. 2008). Second, limb buds lacking the function of *Hoxa13* showed a slight distalization of *Hoxa11* expression, as if this latter gene was derepressed in distal cells (Yokouchi et al. 1995a; Post and Innis 1999). The additional removal of *Hoxd13* function clearly enhanced this phenomenon (Sheth et al. 2014; Woltering et al. 2014). Furthermore, the same effect was observed upon *Hoxd* gene expression, whose transcription was equally gained in more distal domains (Sheth et al. 2014), suggesting that HOX13 proteins may indeed globally repress the T-DOM-dependent transcription of *Hoxd* genes and thus terminate forearm-specific patterning instructions.

Here, we investigated the possibility that the HOX13 proteins have a direct impact on the transcription of *Hoxd* genes to repress their transcription in the digital plate, thereby establishing the expression boundary between the proximal and distal limb domain, leading to the positioning of the wrist. We show that HOXA13 binding is enriched at the *HoxD* locus within both TADs. In the T-DOM, HOXA13 exerts a negative effect by switching off transcriptional activity, an effect genetically enhanced by the presence of HOXD13. In contrast, the binding of HOXA13 to the regulatory islands within the C-DOM is required to sustain transcription in developing digits. This antagonistic effect of HOX13 proteins on the two TADs flanking the *HoxD* cluster explains why these two regulatory landscapes are never active concomitantly in the same cells during limb development. It also gives a molecular basis to this important switch in regulations, which is necessary for the emergence and positioning of the wrist. We discuss the relevance of these observations in the evolutionary framework of the fin-to-limb transition.

## Results

**Figure 1.**
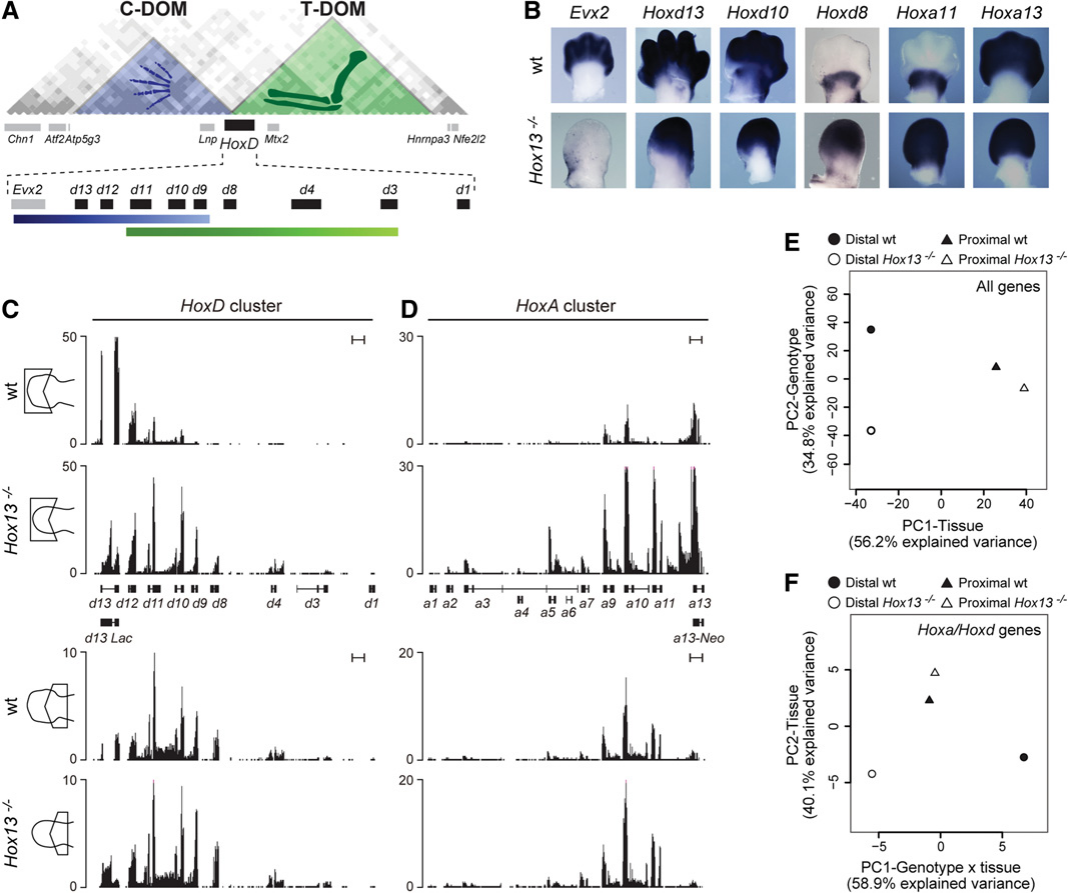
Loss of HOX13 functions affects limb patterning and proximal to distal identities. (*A*) Hi-C data adapted from Dixon et al. (2012) showing the positions and distribution of the two TADs (C-DOM and T-DOM) flanking the *HoxD* cluster. Black and gray boxes represent *Hoxd* and unrelated genes, respectively. The blue and green graded lines *below* indicate the two sets of *Hoxd* genes, which are expressed in either the distal domain (blue) or the proximal domain (green). C-DOM and T-DOM are depicted with the same colors to indicate the presence of the corresponding enhancer sequences. (*B*) In situ hybridization analysis showing the expression of different genes (indicated at the *top*) in the forelimbs of either wild-type (wt) or *Hoxa13*^−/−^*; Hoxd13*^−/−^ (*Hox13*^−/−^) double-mutant specimens at E12.5–E13. While some genes maintain their transcription profiles, others are either lost or gained in the distal part. (*C*,*D*) Transcription profiles of the *HoxA* and *HoxD* clusters in microdissected distal and proximal forelimb domains from either wild-type or *Hox13*^−/−^ mutant specimens at E12.5. Bar, 5 kb. While the profiles remain the same in control and mutant proximal domains, clear differences are observed in the distal limb. (*E*,*F*) Principal component analysis (PCA) of the transcriptomes obtained from proximal and distal forelimb samples dissected out from either wild-type or *Hox13*^−/−^ mutant specimens. The analysis was carried out by using the log_2_ transformed FPKM (fragments per kilobase per million mapped fragments) values of either all autosomal genes (*E*) or the genes present only in the *HoxA* and *HoxD* cluster (*F*). In both cases, the *Hox13*^−/−^ distal domain (empty circle) does not cluster with either the wild-type counterpart (filled circle) or the wild-type proximal (filled triangle) domain. The separation between wild-type and *Hox13*^−/−^ distal limb samples is visible on the second PCA axis when considering all autosomal genes (*E*) and on the first axis when considering only genes located within the *HoxA* and *HoxD* clusters (*F*).

The *HoxD* cluster is flanked by two large gene deserts (T-DOM and C-DOM), which match the extent of two TADs (Fig. 1A; Dixon et al. 2012). The telomeric T-DOM contains at least two enhancers (CS39 and CS65), which regulate the transcription from *Hoxd8* to *Hoxd11* into a proximal limb domain (Andrey et al. 2013). The centromeric C-DOM contains at least six enhancers, which participate in the control of *Hoxd13* to *Hoxd9* transcription into presumptive digits (Montavon et al. 2011). While mice mutant for *Hoxd13* display ill-formed digits (Dolle et al. 1993), double mutants for both *Hoxd13* and *Hoxa13* suffer from severe agenesis of the most distal limb pieces (Fromental-Ramain et al. 1996; Kmita et al. 2005). However, such mutant limbs at embryonic day 12.5 (E12.5) still display a distal cellular field, which clearly resembles a hand plate although smaller in size (Fig. 1B). We asked whether this distal domain was a reduced autopod in molecular terms or instead whether the double loss of function of group 13 genes had lead to an extension of the zeugopodial (forearm) identity due to the absence of distal determinants.

### Genetic identity of Hox13 double-mutant limbs

We performed RNA sequencing (RNA-seq) analysis from proximal and distal developing wild-type forelimbs at E12.5 and selected a set of genes showing at least a threefold biased expression level between the proximal and distal domains, referred to here as “proximal” and “distal” genes. We looked at their expression in *Hoxa13*^−/−^*;Hoxd13*^−/−^ double-mutant limbs (termed here *Hox13*^−/−^) at E12.5 and obtained somewhat conflicting results. The distal-most aspect of limb buds lacking both HOXA13 and HOXD13 functional proteins due to insertional mutagenesis still contained *Hoxa13* and *Hoxd13* transcripts, suggesting the persistence of a distal identity (Fig. 1B). Likewise, expression of *Hoxd10* and *Hoxd11* was maintained distally (Fig. 1B,C), as well as those of other distal genes such as *Prrx2* and *Lhx2* (Supplemental Fig. S1; Rincon-Limas et al. 1999). On the other hand, some proximal gene expression, including *Shox2* and *Hoxa11* (Neufeld et al. 2014), was clearly gained within the distal domain, suggestive of an extension of proximal identity into the most distal limb aspect (Fig. 1B,D; Supplemental Fig. S1). Also, a number of genes expressed in the distal limb, including *Dbx2*, *Evx2*, and *Aldh1a2*, showed reduced levels of mRNAs in distal cells of *Hox13*^−/−^ mutant limbs (Fig. 1B; Supplemental Fig. S1; Shou et al. 2013).

To have a more quantitative assessment, we microdissected proximal and distal pieces from *Hox13*^−/−^ mutant limbs at E12.5 and produced RNA-seq data sets. Expectedly, the analysis of both *HoxA* and *HoxD* clusters revealed no striking difference between control and *Hox13*^−/−^ mutant proximal limbs; i.e., the domain where neither *Hoxa13* nor *Hoxd13* is normally expressed (Fig. 1C,D). In the *Hox13*^−/−^ mutant distal limb, however, the profiles were distinct from their controls and looked more similar to the proximal limb profiles. In the case of *HoxD*, *Hoxd8*, *Hoxd9*, *Hoxd10*, and *Hoxd11* mRNAs were gained, resembling the situation seen in the proximal limb (Fig. 1C). The up-regulation of *Hoxd8* in the distal domain of mutant limbs was unexpected, since this gene is normally not strongly regulated by the digit enhancers located within the C-DOM. Likewise, *Hoxa11* was gained in the *Hox13*^−/−^ mutant distal limb, as shown by in situ hybridization (Sheth et al. 2014; Woltering et al. 2014), as well as other more proximal genes like *Hoxa5* and *Hoxa7* (Fig. 1B,D). Therefore, the expression profiles in the distal limb domain of *Hox13*^−/−^ mutants were distinct from their control counterparts and generally more related to those of control proximal limbs. Surprisingly, however, the global expression levels of both *Hoxd* and *Hoxa* genes in *Hox13*^−/−^ distal limb domains were higher than in the control proximal domain (Fig. 1B–D; Supplemental Fig. S1), suggesting that HOX13 proteins negatively regulate the mRNA levels of other *Hox* genes. At the *HoxD* locus, the *Lnp* and *Evx2* genes, which are localized within the C-DOM on the anti-*Hox* DNA strand and normally are coexpressed with *Hoxd13* in the distal limb, were significantly down-regulated in the *Hox13*^−/−^ mutant condition (Supplemental Fig. S2).

To assess more generally whether these differences in *Hox* expression profiles reflected a transformation of the distal domain identity toward a more proximal fate, we compared the transcriptomes using a principal component analysis (PCA). When all autosomal genes were considered (Fig. 1E), the majority of the variability observed among our samples (56.2%) was attributable to the proximal versus distal identity of the analyzed domains. Along this axis, the control and the *Hox13*^−/−^ mutant proximal limb data sets clustered together and were separated from both the control and *Hox13*^−/−^ mutant distal data sets, indicating that in the *Hox13*^−/−^ mutant situation, the distal part of the limb did not simply acquire a proximal identity. The control and *Hox13*^−/−^ mutant distal domains were nevertheless clearly differentiated along the second axis of the PCA (which accounted for 35% of the total variability), supporting the idea that both functions of *Hox13* genes are required for the specification of normal autopods. In contrast, the proximal domains from control and *Hox13*^−/−^ mutant limbs showed very little difference, as expected from the lack of function of *Hox13* genes in this territory.

When the PCA was restricted to the genes, transcripts, and long noncoding RNAs (lncRNAs) present on both strands in the *HoxA* and *HoxD* clusters only, most of the variability across samples resulted from the difference between the control and *Hox13*^−/−^ mutant distal limb domains (Fig. 1F). The first PCA component clearly separated the two distal limb samples, whereas both proximal limb samples clustered together at an intermediate position on the same axis. However, proximal and distal limb identities were still separated along the second PCA component, supporting the observation that the “*Hox* identity” of the *Hox13*^−/−^ mutant distal limb territory was not transformed into a proximal identity. These results suggested that both *Hoxa* and *Hoxd* genes were more affected by the lack of HOX13 proteins than the bulk of other genes expressed in this distal domain, thus raising the possibility that HOX13 function might be involved in *Hox* gene regulation within this domain.

### Lack of *HOX13* proteins has an impact on global HoxD regulation

*Hoxd* genes up-regulated distally in the absence of HOX13 proteins were precisely those that are strongly coregulated in the wild-type proximal domain (Andrey et al. 2013), suggesting that in the *Hox13*^−/−^ mutant limbs, the digit enhancers within the C-DOM had changed their realm of action to become able to regulate genes located at a more telomeric position, such as *Hoxd8.* Alternatively, the telomeric forearm-specific enhancers, normally at work in the proximal domain only, may have lost this restriction in *Hox13*^−/−^ mutant limbs to continue to exert their regulatory function into the most distal cells. To discriminate between these two scenarios, we used different alleles where the digits enhancers could no longer regulate their *Hoxd* targets.

**Figure 2.**
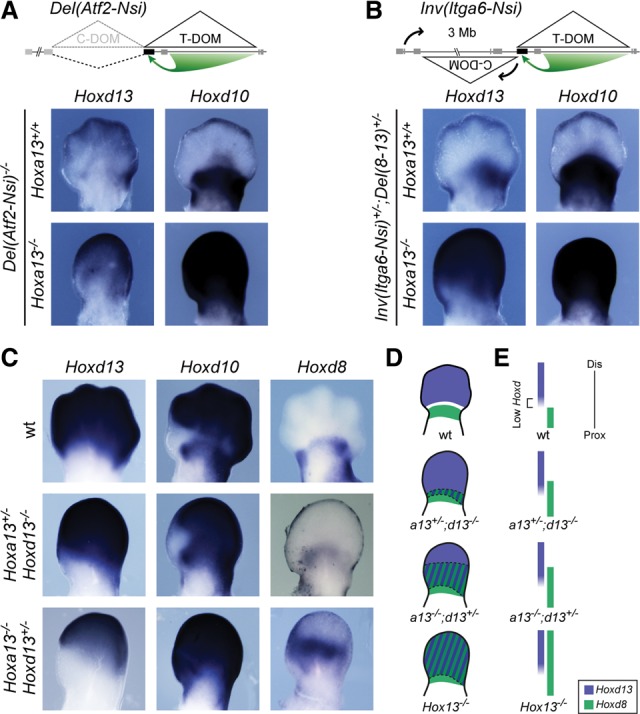
T-DOM drives both proximal and distal expression of *Hoxd* genes in double *Hox13* mutant forelimbs. (*A*,*B*) In situ hybridization analysis of E12.5 forelimbs showing the expression of both *Hoxd10* and *Hoxd13* in two different mutant stocks where the C-DOM is disconnected from the cluster. (*A*, *top*) In the *HoxD*^*Del(Atf2-Nsi)*^ line the C-DOM is removed through an ∼1-Mb large deletion (dashed line). (*B*, *top*) In the *HoxD*^*Inv(Itga6-Nsi)*^ line, the C-DOM is repositioned several megabases away from the *HoxD* cluster via a large inversion (black arrows). In both cases, expression of *Hoxd10* and *Hoxd13* is lost in the distal domain. Expectedly, *Hoxd10* remains transcribed proximally, under the control of the T-DOM. (*A*,*B*, *bottom*) However, when the function of *Hoxa13* was further removed, expression of both *Hoxd10* and *Hoxd13* was restored in the distal domain even in the absence of the C-DOM regulation. (*C*) In situ hybridization analysis of E12.5 forelimbs showing the expression of *Hoxd8*, *Hoxd10*, and *Hoxd13* in embryos carrying various combinations of mutated alleles (indicated at *left*). (*D*,*E*) A schematic representation shows the C-DOM-driven distal expression domain in blue (e.g., *Hoxd13*) and the T-DOM-driven proximal expression domain in green (e.g., *Hoxd8*). In wild type, the domain of low *Hoxd* expression between *Hoxd13*- and *Hoxd8*-expressing regions is represented in white in *D* and with a bracket in *E*. In *Hox13*^−/−^ mutant limbs, the T-DOM controls all *Hoxd* genes up to the most distal part of the limb (merged domain shown by stripes in *D*), leading to an ill-defined genetic identity. In such a situation, a presumptive mesopodium (white in *D*, can no longer appear.

The first allele was a large *HoxD*^*Del(Atf2-Nsi)*^ deletion, which removes the entire C-DOM [Fig. 2A, *Del*(*Atf2-Nsi*)]. In this deletion, expression of *Hoxd13* to *Hoxd10* was expectedly abolished in the distal domain at E12.5 due to the lack of the appropriate enhancers (Fig. 2A, top), whereas *Hoxd10* was still expressed in the proximal part in response to the intact telomeric enhancers (Fig. 2A). However, the combined abrogation of *Hoxa13* function induced restoration of both *Hoxd13* and *Hoxd10* expression in the distal parts of limbs at E12.5 (Fig. 2A, bottom). As anticipated, the distribution of *Hoxa13* transcripts did not change in these mutants (data not shown). From this experiment, we concluded that *Hoxd13* and *Hoxd10* could be transcribed in most distal limb cells in the absence of digit enhancers located within the deleted C-DOM.

We validated this observation with a second allele where C-DOM was relocated several megabases away from its target *Hoxd* genes through an inversion (Fig. 2B). As for the deleted allele, this *HoxD*^*Inv(Itga6-Nsi)*^ inversion led to the absence of any *Hoxd13* to *Hoxd10* transcripts in the distal domain at E12.5 [Fig. 2B, *Inv*(*Itga6-Nsi*); Tschopp and Duboule 2011] due to the increased enhancer-to-promoter distance. Here again, however, the additional abrogation of *Hoxa13* function restored expression in the distal domain at E12.5 (Fig. 2B, bottom), as if digit enhancers were at work. From these results, we concluded that the restored distal expression in these two double mutants was controlled by the T-DOM regulation rather than by the usual digit enhancers located in C-DOM. This implied that T-DOM has the intrinsic capacity to drive *Hoxd* gene transcription into the digit domain, a capacity normally inhibited by HOX13 proteins. In both the *Del(Atf2-Nsi)* and *Inv(Itga6-Nsi)* alleles, the constitutive interactions observed between *Hoxd13* and the C-DOM (Montavon et al. 2011) had disappeared and were likely replaced by looser interactions with the new genomic neighborhoods (Andrey et al. 2013), thus allowing the establishment of unusual contacts between *Hoxd13* and T-DOM enhancers. These differences in the new genomic neighborhoods due to either the deletion or the inversion of the C-DOM explained the slight variations in the level of ectopic *Hoxd13* transcripts now controlled by the T-DOM.

This gain of *Hoxd* gene expression in the distal domain scored in the absence of HOX13 proteins also occurred in *Hoxa13*^−/−^*;Hoxd13*^+/−^ mutant limbs at E12.5, indicating a dosage effect. However, this gain was much weaker, as seen with *Hoxd8* transcripts, which were detected more distally than in control limbs yet not in the most distal aspect of the limbs (Fig. 2C). The restoration of *Hoxd10* distal expression in these different mutant configurations produced a single extended domain instead of the two “classical” proximal and distal domains expected from the switch between T-DOM and C-DOM regulation (Figs. 1B, 2). Accordingly, the zone of low *Hoxd* expression separating the two domains (Fig. 2D,E top, white zone) had disappeared to generate a continuous expression domain (Fig. 2D,E, bottom).

### Binding of *HOX13* within both the C-DOM and T-DOM

**Figure 3.**
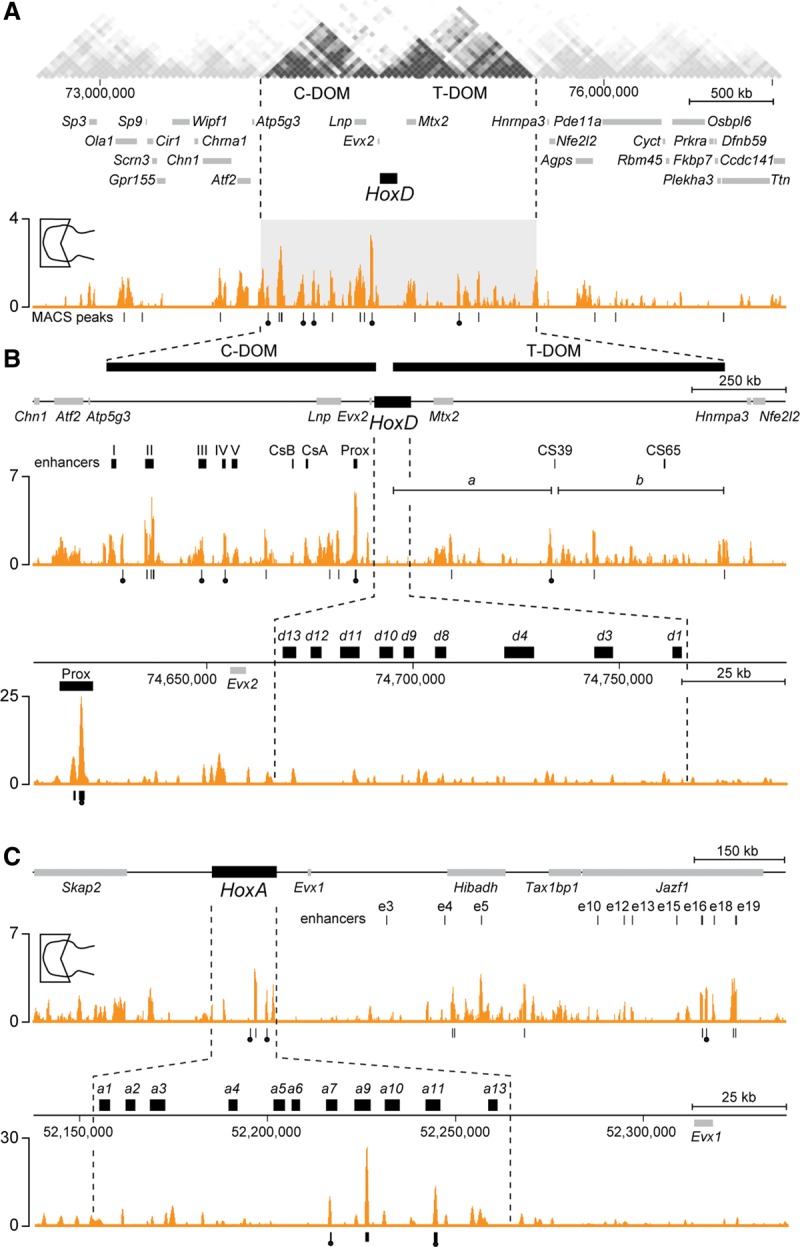
Enrichment of bound HOXA13 protein in both T-DOM and C-DOM regulatory landscapes. (*A*) HOXA13 ChIP-seq data in the distal domain of the forelimb at E12.5. Enrichment (*Y*-axis) is shown as the difference of the normalized number of reads between ChIP and input samples. Peaks identified by MACS analysis are represented as vertical traits *below* the HOXA13-binding profile. Peaks identified by MACS in both the mouse and the chick data sets are marked with filled circles (see also in Supplemental Fig. S3). (*Top*) A profile over 4 Mb of DNA shows enriched binding sites for HOXA13 in both the C-DOM and the T-DOM (Dixon et al. 2012). (*B*) Successive close-up views of bound HOXA13 in either both the C-DOM and the T-DOM or the *HoxD* cluster. Bound HOXA13 was found at previously identified regulatory regions, such as islands II, III, and IV and Prox (*top* track), whereas no binding was observed in the *HoxD* cluster (*bottom* track). (*Top* track) Regions “a” and “b” represent the two sub-TADs previously described within the T-DOM. (*C*, *top* track) The analysis of HOXA13 binding at the *HoxA* locus also revealed a specific enrichment at previously characterized *HoxA* digit regulatory elements such as e16 and e19 (Berlivet et al. 2013). (*Bottom* track) In contrast to *HoxD*, significant binding was scored within the cluster at the *Hoxa7*, *Hoxa9*, and *Hoxa11* loci.

To investigate whether this repressive effect of HOX13 proteins was due to a direct interaction, we performed ChIP-seq (chromatin immunoprecipitation [ChIP] combined with high-throughput sequencing) analysis using an antibody against HOXA13. In E12.5 wild-type distal limb cells, HOXA13 binding was enriched around the *HoxD* locus at large with a series of peaks telomeric to the *HoxD* cluster, as identified by using model-based analysis of ChIP-seq (MACS) (Fig. 3A; Zhang et al. 2008). Interestingly, these signals extended approximately over the length of the T-DOM (Fig. 3A), supporting a potential direct negative effect of HOXA13 protein over T-DOM limb enhancers. In particular, a significant peak was scored near the CS39 proximal enhancer (Fig. 3B; Andrey et al. 2013). Some of these signals were also observed at cognate genomic positions when stage HH28 chicken distal limbs (Hamburger and Hamilton 1992) were used in similar ChIP-seq experiments, further validating the significance of peaks (Fig. 3, filled circles below the profiles; Supplemental Fig. S3).

Unexpectedly, however, several strong HOXA13 peaks were also found centromeric to *HoxD*. Again, signals were detected essentially throughout the length of the C-DOM, with peaks observed precisely over most of the previously defined islands necessary for digit regulation (Montavon et al. 2011) in both mouse and chick limbs (Fig. 3B; Supplemental Fig. S3). In contrast to the situation in the T-DOM, this latter observation suggested that in the C-DOM, HOXA13 might be required to maintain or re-enforce the digit regulation. As in the case of the T-DOM, these contacts were not seen in the proximal limb (Supplemental Fig. S4) or much attenuated, likely due to a weak contamination in dissection. Alternatively, some residual HOX13 protein may be present in low amounts due to invading muscle precursor cells (Yamamoto et al. 1998). Interestingly, no HOXA13 binding was scored within the mouse *HoxD* cluster itself (Fig. 3B).

We also investigated the binding of HOXA13 at the *HoxA* locus, which contains similar limb regulatory sequences (Lehoczky and Innis 2008; Berlivet et al. 2013). There again, enrichments were found over the regulatory regions that control *Hoxa13* during digit development, in particular at the positions of the e16 and e19 digit enhancers (Fig. 3C; Berlivet et al. 2013). In addition and unlike for *HoxD*, HOXA13 binding was observed within the *HoxA* cluster itself in both the mouse and chick samples at the *Hoxa11*, *Hoxa9*, and *Hoxa7* loci (Fig. 3C; Supplemental Fig. S3).

### The absence of a regulatory switch in Hox13 mutant limbs

**Figure 4.**
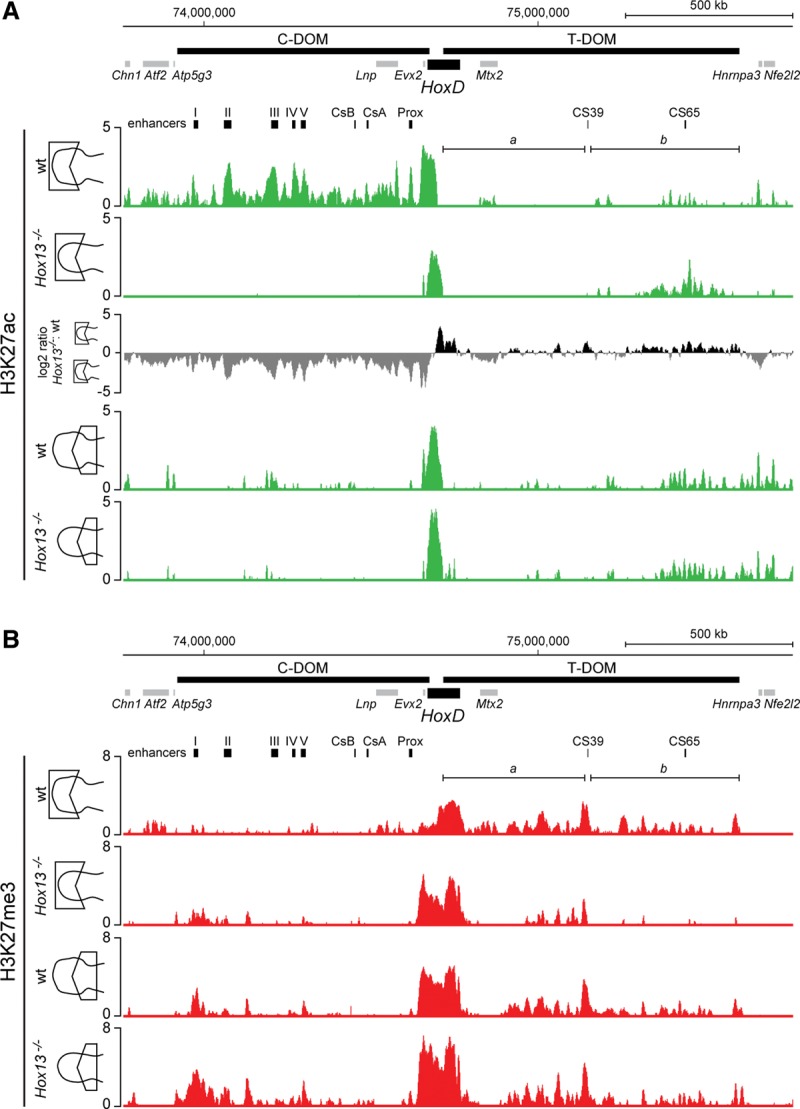
Lack of HOX13 function prevents the regulatory switch from the T-DOM to the C-DOM. (*A*) Comparison of H3K27ac profiles in the distal and proximal parts of forelimbs obtained from either wild-type or *Hox13*^−/−^ double-mutant specimens at E12.5. While H3K27ac peaks are distributed throughout the C-DOM in the control track (*top*), distal *Hox13*^−/−^ mutant limbs displayed an almost complete absence of H3K27ac signals (second track). In the latter, however, signals appeared within a region of the T-DOM, similar to the profiles observed in the proximal sample (tracks *below*). The log_2_ ratio of the normalized number of reads between H3K27ac of *Hox13*^−/−^ mutant and wild-type distal limbs is shown by positive (black) and negative (gray) values, respectively. (*B*) Distribution of H3K27me3 marks in distal and proximal forelimbs of either wild-type or *Hox13*^−/−^ mutant specimens at E12.5. In the distal part of the *Hox13*^−/−^ mutant, H3K27me3 enrichment of the T-DOM was dramatically reduced, in particular within subdomain “b,” corresponding to the increased enrichment of H3K27ac (shown in *A*). In contrast, H3K27me3 enrichment around the C-DOM-located islands I and II was slightly increased, in agreement with the loss of H3K27ac (shown in *A*). Enrichment (*Y*-axis) is shown as the log_2_ ratio of the normalized number of reads between ChIP and input samples, except for the third track in *A*. The localizations of genes and regulatory islands are shown at the *top* of each panel.

We assessed the functional states of both the T-DOM and the C-DOM in the absence of HOX13 proteins by comparing the distribution of chromatin modifications between wild-type control and double *Hox13*^−/−^ mutant proximal and distal limbs at E12.5 (Fig. 4). We looked at both the acetylation of H3K27 (H3K27ac; a mark associated with enhancer and transcriptional activity) and the trimethylation of the same residue (H3K27me3; a modification associated with polycomb-dependent silencing). In the wild-type distal sample, H3K27ac was enriched over the C-DOM, illustrating the intense activity of this regulatory landscape during digit development, with robust peaks over the previously defined islands (Fig. 4A, track 1). In contrast, the low level of this mark over the T-DOM reflected its silent state in such distal cells (Andrey et al. 2013). In the proximal limb, the opposite profile was scored, with only traces of H3K27ac over the C-DOM and a concentration of these modified histones at the telomeric end of the T-DOM, corresponding to one of the two subinteraction domains contained within the T-DOM (Fig. 4, bracket b). At E12.5, indeed, the level of H3K27ac detected in these proximal cells started to decrease while remaining high in the latter part of the telomeric landscape (Andrey et al. 2013).

In *Hox13*^−/−^ mutant distal limbs, no enrichment of H3K27ac was detected over the entire centromeric region (Fig. 4A, track 2), indicating that this regulatory landscape had not been activated. However, H3K27ac marks were clearly enhanced in the T-DOM precisely at the place where these marks were scored in proximal cells; i.e., in cells implementing the T-DOM regulation (Fig. 4A, bracket b). As expected, the H3K27ac profiles were comparable between mutant and control proximal limbs, which do not express any *Hox13* gene (Fig. 4A, tracks 3, 4). These observations suggested that in distal limb cells lacking HOX13 function, the T-DOM regulation was no longer interrupted and continued to exert its regulatory activity, whereas the C-DOM regulation was either not implemented at all or at least not maintained after an initial burst.

We verified this conclusion by producing the profile of H3K27me3, which antagonizes the acetylation of the same residue (Tie et al. 2009). Here again, the profiles of control and *Hox13*^−/−^ proximal limb domains did not significantly differ from one another (Fig. 4B, bottom lines). Signals were detected in the T-DOM, in particular in the region where few H3K27ac marks were scored (Fig. 4A,B; bracket a). In contrast, signals were weaker in the region where H3K27ac was detected (Fig. 4A,B; bracket b), indicating the progressive termination of T-DOM function as normally observed in E12.5 proximal limbs (Andrey et al. 2013). In control distal cells, these repressive marks equally accumulated over the T-DOM after it had switched off. Strikingly, this accumulation was not scored in the *Hox13*^−/−^ mutant distal domain (Fig. 4B, brackets b), further indicating that the telomeric regulation was kept abnormally active in distal cells.

The distribution of both H3K27ac and H3K27me3 modifications over the *HoxD* cluster itself supported this interpretation. The H3K27ac profile in the control proximal domain mostly encompassed *Hoxd8* to *Hoxd11*, with significant signals also detected over *Hoxd12*, *Hoxd13*, and *Evx2* (Supplemental Fig. S5). In the *Hox13*^−/−^ specimen, this profile was globally conserved, with the exception of *Hoxd13* and *Evx2* displaying somewhat weaker signals. In the distal limb domain, however, the H3K27ac profile was virtually identical to that of the control proximal domain (Supplemental Fig. S5), with a strong reduction over the *Evx2*–*Hoxd13* region and a gain for the *Hoxd8* and *Hoxd9* regions, which are normally strong targets of T-DOM regulation.

In wild-type proximal limbs, H3K27me3 marks showed a bimodal distribution with strong coverage of both the 3′ and 5′ extremities of the cluster; i.e., those subgroups of genes not transcribed at this stage. In between, the active *Hoxd8* to *Hoxd11* region contained fewer of these marks. On the other hand, the control distal domain was heavily decorated with H3K27me3 from *Hoxd1* to *Hoxd8*, whereas the part of the cluster responding to the C-DOM regulation in digits was mostly devoid of it (from *Hoxd9* to *Hoxd13*) (Supplemental Fig. S5). In the mutant condition, this latter profile drastically changed to appear like the control proximal profile, suggesting again that the C-DOM regulation was inactive, in particular considering the robust H3K27me3 coverage of *Hoxd13*, *Hoxd12*, and *Evx2* (Supplemental Fig. S5B, track 2). In mutant distal cells, however, the H3K27me3 coverage over the *Hoxd12–Hoxd13* region was less compact than in mutant proximal cells (Supplemental Fig. S5), coinciding with the weak distal expression of *Hoxd13* unexpectedly controlled by T-DOM regulation (see Fig. 2A,B). In contrast, H3K27me3 coverage was comparable in the *Hoxd1* to *Hoxd4* region of distal versus proximal mutant cells.

The distribution of H3K27ac and H3K27me3 marks over the *HoxA* cluster in control and mutant cells also suggested that a “proximal” type of regulation was maintained in distal cells lacking HOX13 function. This was best illustrated by both an increased acetylation of the *Hoxa1* to *Hoxa7* region and a decreased acetylation of the *Hoxa11* to *Hoxa13* region in mutant distal cells when compared with control, thus resembling the H3K27ac profiles of both control and mutant proximal cells (Supplemental Fig. S6).

### *HOX13*-dependent modifications in three-dimensional (3D) interactions

**Figure 5.**
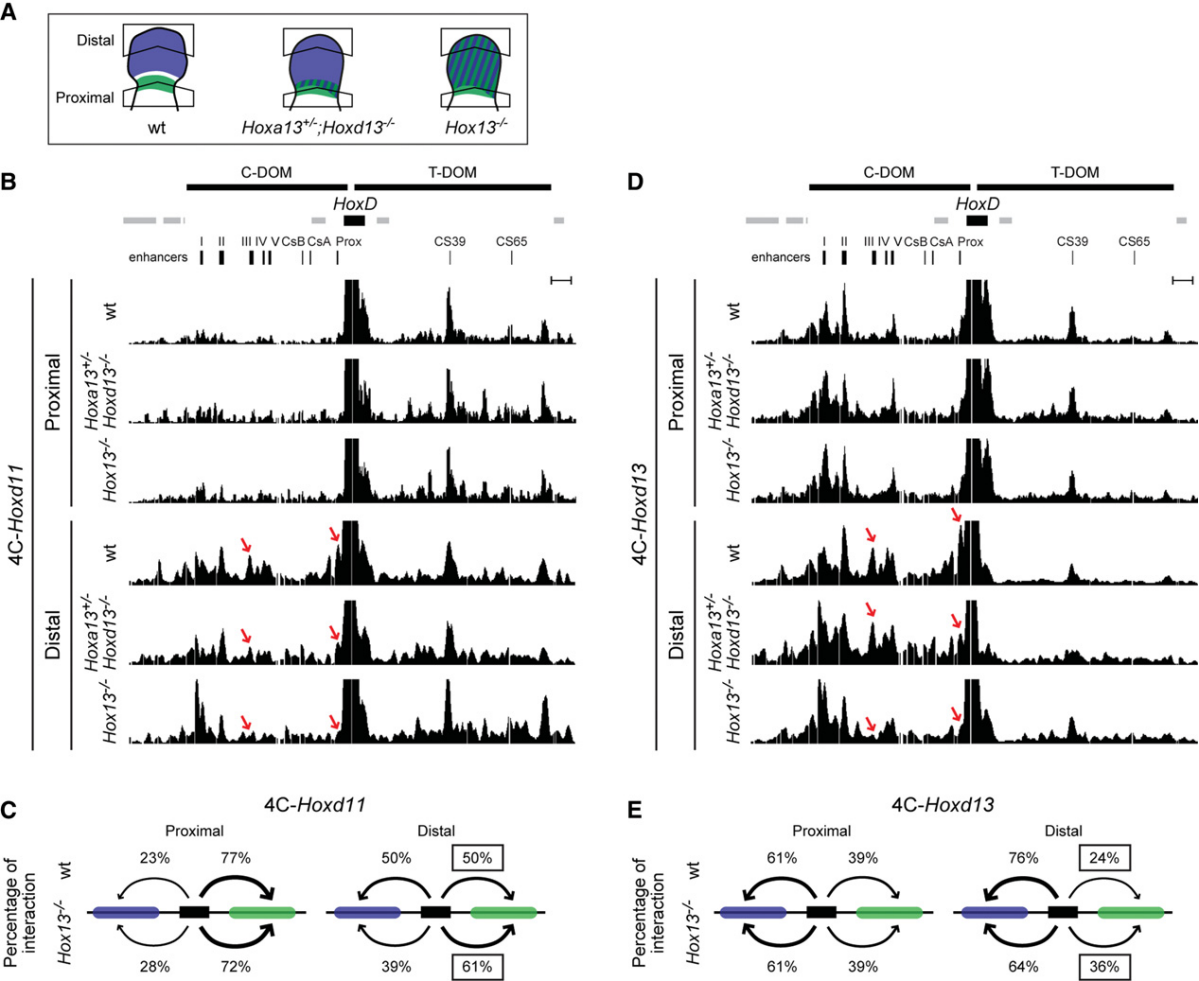
Reallocation of interactions from the C-DOM to the T-DOM in limbs lacking HOX13 function. (*A*) Schemes showing the dissection strategies used for sampling. The color code is as in Figure 2D. (*B*,*D*) 4C-seq tracks representing contacts established by *Hoxd11* (*B*) and *Hoxd13* (*D*) in proximal and distal limb samples (indicated at the *top*) of either wild-type or various *Hox13* mutant combinations at E12.5 (indicated at the *left*). The extents of both the C-DOM and the T-DOM are shown as thick black lines at the *top* of *B*, together with the positions of genes. The previously described regulatory elements are indicated *below* (in black). The red arrows in the distal samples point to specific contacts detected between genes normally expressed in the distal part under the control of the C-DOM (Andrey et al. 2013). These interactions with island III and *Prox*, which are hallmarks of the C-DOM operating in limbs, are no longer detected in distal samples lacking all HOX13 function. (*C*,*E*) Schemes representing the percentages of 4C contacts for either *Hoxd11* (*C*) or *Hoxd13* (*E*) with the C-DOM (blue rectangle) and the T-DOM (green rectangle) in the various samples. The *HoxD* cluster is shown as a black rectangle. While virtually no difference was observed in the proximal samples, both *Hoxd11* and *Hoxd13* in the *Hox13*^−/−^ double-mutant distal sample lost contacts with the C-DOM and gained contacts with the T-DOM, in agreement with their transcription now controlled by this latter regulatory landscape.

The regulation of *Hoxd13*-to-*Hoxd9* transcription by C-DOM enhancers in developing digits involves strong physical interactions. Among these contacts, those with the island III and *Prox* sequences are hallmarks of *Hoxd13* transcriptional activation (Montavon et al. 2011). By using circularized chromosome conformation capture (4C) coupled with next-generation sequencing (4C-seq), interactions between *Hoxd13* or *Hoxd11* and these two sequences were indeed scored in all cases where the material was derived from developing digits but absent from control negative tissues, which display only constitutive nonproductive contacts with *Hoxd13* (Andrey et al. 2013; Lonfat et al. 2014). We thus looked for these contacts in double-mutant distal limbs at E12.5 as an ultimate assessment of the nonfunctionality of the C-DOM in these cells. Because of the reduced size of the distal domain in double *Hox13* mutant limbs (see Figs. 1, 2), we restricted our microdissections to the most proximal and most distal pieces of the developing limbs to prevent cross-contamination between cells (Fig. 5A).

In control proximal cells, *Hoxd11* mostly contacted the T-DOM, with a particularly strong interaction with region CS39. In *Hox13*^−/−^ double mutants, the interaction profiles were globally comparable (Fig. 5B, top). In distal cells, however, the *Hoxd11* interaction profile changed drastically in mutant versus control samples (Fig. 5B, bottom), as the reported shift in contacts from the T-DOM toward the C-DOM (Andrey et al. 2013) was no longer observed. A quantification of contacts indicated 77% of telomeric contacts for *Hoxd11* in control proximal cells and 50% in distal cells, showing that *Hoxd11* had reallocated almost 30% of its contacts toward the C-DOM in digit cells (Fig. 5C). Within the C-DOM, interactions were scored with the islands III and *Prox* sequences (Fig. 5B, red arrows; enlargement in Supplemental Fig. S7), indicative of an active transcriptional state.

In contrast, mutant distal cells did not show these interactions (Supplemental Fig. S7). Instead, contacts were re-enforced within the T-DOM, further indicating that *Hoxd11* had remained regulated by this landscape rather than shifting toward the C-DOM. The transcriptional inactivity of C-DOM regulatory sequences was corroborated by the interaction profile of *Hoxd13* (Fig. 5D). In control distal cells, *Hoxd13* showed the expected robust contacts with the C-DOM, including strong interactions with both region III and *Prox* (Fig. 5D, red arrows). In the double *Hox13* mutant limb cells, however, while the bulk of interactions remained over the C-DOM, as in control negative cells like brain tissue (Montavon et al. 2011), the specific interactions with region III and *Prox* had virtually disappeared along with the transcriptional off state of the C-DOM. Interestingly, contacts established by *Hoxd13* with the T-DOM were slightly increased (from 24% to 36%) (Fig. 5E), in agreement with the weak expression of this gene in distal mutant cells (Fig. 1B,C). As expected, the interaction profiles of *Hoxd13* in control and mutant proximal cells were comparable.

We also assessed the impact of HOX13 dosage on the control of this regulatory switch by looking at the interaction profiles of intermediate allelic combinations. We analyzed both *Hoxa13*^−/−^*;Hoxd13*^+/−^ (data not shown) and *Hoxa13*^+/−^*;Hoxd13*^−/−^ (Fig. 5) mutant limbs. In these mutants, autopods were also severely impaired, yet a distal domain devoid of the *Hoxd8* transcript was still identified (Fig. 2C,D), unlike in full *Hox13*^−/−^ embryos (Figs. 1B, 2D), indicating that the switch had been achieved at least partially. Accordingly, we observed only slight and mostly quantitative modifications in the interaction profiles. For example, weaker yet present contacts between both *Hoxd11* and *Hoxd13* and region III and *Prox* were scored in mutant distal cells, showing that the C-DOM was still at work, although with a lower transcriptional capacity. Although a single dose of HOX13 protein was sufficient to sustain the C-DOM regulatory program in both cases, it failed to fully implement this complex regulation. This quantitative effect was similar when the last *Hox13* copy left was either *Hoxa13* or *Hoxd13* (Fig. 5; data not shown), suggesting a close to equal function of both proteins in this context. From these experiments, we concluded that both proteins cooperate in a dose-dependent manner to repress the activity of the T-DOM and/or maintain transcription driven by the C-DOM.

### Distal repression of the T-DOM is not targeted to enhancer sequences

While the pattern of HOXA13 occupancy within the C-DOM was rather specific to known regulatory islands, peaks in the T-DOM were mostly concentrated in subdomain “b” (Fig. 3B), where most of the *Hoxd11* contacts were gained when 4C-seq was carried out with double-mutant distal limbs (Fig. 5B). However, HOXA13 binding was not observed in either of the two known CS39 and CS65 proximal limb enhancers. Instead, the most significant HOXA13 signal was located near CS39, matching the CS38 sequence, which contains the transcription start sites of the two opposite lncRNAs: *Hotdog* and *Twin of Hotdog* (Delpretti et al. 2013). This indicated that HOX13 repression of T-DOM regulation may not involve direct binding to forearm-specific enhancers.

**Figure 6.**
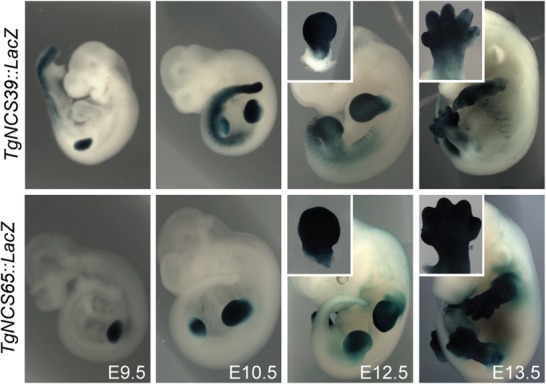
The T-DOM CS39 and CS65 sequences are global rather than proximal limb enhancers. Mouse stable transgenic lines carrying a *LacZ* reporter construct under the control of either the *CS39* (*top*) or the *CS65* (*bottom*) enhancers. In both cases, expression was scored throughout the developing limbs from the budding stage at E9.5 (*left*) to at least E13.5 (*right*).

To test this hypothesis, we produced lines of *LacZ* reporter transgenic mice carrying random integrations of either the CS39 or the CS65 forearm-specific enhancers, reasoning that such sequences may escape repression in distal limbs when placed outside of the T-DOM context due to the absence of direct HOXA13 binding. In both cases, after an initial phase of expression in the proximal limb bud, *LacZ* staining extended up to the most distal limb aspects at E12.5 (Fig. 6), suggesting that the transgenes had escaped the HOXA13-dependent repression normally associated with T-DOM. These experiments showed that both CS39 and CS65 enhancers have the capacity to drive expression of a reporter gene throughout the developing limb buds. However, this capacity is normally inhibited in distal limb cells due to the action of HOX13 proteins over the T-DOM.

## Discussion

### TAD switching and the wrist boundary

The presumptive wrist region corresponds to an area of the developing limb bud where *Hoxd* gene expression is minimal. As a result, the ossification of cartilage condensations does not process along the proximal to distal axis (Gonzalez-Martin et al. 2014), leading to the nonelongated mesopodial bone morphologies (Villavicencio-Lorini et al. 2010). This cellular zone devoid of *Hoxd* gene transcripts results from the bimodal regulatory strategy investigated in this study. Here, we show that the boundary between the wrist and the future forearm is fixed by the activation of *Hox13* genes, which repress the transcription of *Hoxd* genes, likely through direct binding within the T-DOM. The HOXA13 protein switches off the T-DOM regulation and hence prevents proximal *Hoxd* genes from being transcribed more distally, including in the wrist region. A complete repression also requires HOXD13 combination, as shown by the fact that either *Hoxa13*^−/−^ or *Hoxd13*^−/−^ single-mutant animals have genuine yet slightly affected wrists (Dolle et al. 1993; Fromental-Ramain et al. 1996), whereas double-mutant limbs do not display any structure reminiscent of this articulation (Fromental-Ramain et al. 1996; Kmita et al. 2005).

### *HOX13* proteins as C-DOM activators?

Our results suggest that HOX13 proteins are essential to fully activate and/or sustain the regulatory activity of the C-DOM. Their combined abrogation leads to the absence of TAD switching, with the C-DOM never becoming detectably active. While the underlying mechanism of action is elusive, our ChIP-seq results indicate that the HOXA13 protein occupies most of the previously determined digit regulatory islands (Montavon et al. 2011). Nevertheless, the *Hoxd13* transcript domain does not perfectly match that of *Hoxa13*, with *Hoxd13* being slightly less proximal (see Woltering and Duboule 2010), indicating that HOXA13 alone is not sufficient to activate *Hoxd13* and that other factors are required to initiate the transcription of *Hoxd* genes in distal cells. Also, *Hoxa13*^−/−^ mutant mice display normal *Hoxd13* transcription. In addition, some of the C-DOM islands bound by HOXA13 establish only constitutive contacts with the target *HoxD* cluster and the HOXA13 signals extended over the size of these islands, usually several kilobases in size. This generally protein-dense aspect is quite distinct from a classical DNA-binding protein profile under the same experimental conditions.

The positive effect of HOX13 proteins in implementing the C-DOM regulation may not be very selective. Instead, the fact that the signals tend to colocalize with all DNA segments in contact with the target *Hoxd13* gene may reflect an impact on the general architecture of the C-DOM itself. Alternatively, the architecture of the C-DOM may favor the recognition of low-affinity binding sites by HOX13 proteins. In any case, it is likely that the same effect will be observed wherever the C-DOM is active. For instance, *Hoxa13*/*Hoxd13* mutant mice lack external genitals (Kondo et al. 1997; Warot et al. 1997), and *Hoxd13* transcription in the genital bud indeed depends on enhancers localized within the same C-DOM (Lonfat et al. 2014).

The HOXA13-binding specificity was controlled by using stage-comparable chicken proximal and distal wing buds. While both the enrichments at the *HoxD* and *HoxA* loci and some conspicuous peaks were conserved between species, some differences were observed. For example, MACS analysis revealed that, while the C-DOM regulatory islands III and IV and *Prox* were bound in both species, region II was bound only in the mouse sample. Likewise, binding to the e16 enhancer at the *HoxA* locus (Berlivet et al. 2013) was detected only in mice. These differences might reflect either intrinsic properties of the antibody or technical issues. They may also underlie some of the genuine differences observed in the expression of these genes during limb bud development in both mammals and birds (Dolle et al. 1989; Nelson et al. 1996).

### *HOX13* proteins as T-DOM repressors?

Within the T-DOM, the pattern of HOXA13 binding was somewhat more diffuse and mostly concentrated on the sub-TAD “b.” This subdomain preferentially interacts with *Hoxd* genes expressed in the forearm region, whereas subdomain “a” contacts the most 3′-located genes on the cluster such as *Hoxd1* (Andrey et al. 2013), which do not have any reported function in limb development. Accordingly, in double *Hoxa13*^−/−^*;Hoxd13*^−/−^ mutant distal limbs, the gain of *Hoxd11* contacts reallocated from the C-DOM to the T-DOM were scored within subdomain “b,” illustrating the correlation between the presence of HOXA13 protein and the transcriptional inactivity of this region in wild-type distal cells. This region was also rich in H3K27ac up to E12.5 in control proximal limbs (Andrey et al. 2013) and abnormally maintained H3K27ac in *Hoxa13*^−/−^*;Hoxa13*^−/−^ mutant distal cells. Therefore, in control distal cells, T-DOM regulation is terminated and repressed in conjunction with the presence of HOX13 proteins bound over the DNA interval, which is normally the most active in contacting the main *Hoxd9*, *Hoxd10*, and *Hoxd11* target genes in proximal cells.

While the negative impact of HOX13 proteins on T-DOM regulation was documented by our genetic and molecular approaches, neither the CS39 nor the CS65 enhancers were bound by HOXA13. However, a robust binding was scored over CS38, a conserved DNA sequence next to CS39. Both CS39 and CS65 enhancers contribute to the transcription of *Hoxd* target genes in the proximal limb, whereas they are normally unable to control the same genes in distal cells (Andrey et al. 2013). When introduced at ectopic genomic positions, these enhancers were nevertheless capable of driving expression distally, showing their intrinsic capacity to control transcription throughout the developing limbs even in presence of physiological amounts of HOXA13 and HOXD13 proteins. We conclude that the restrictive action of these proteins in distal cells is not mediated by direct binding to the enhancers themselves. On the other hand, this distal repression cannot come from the binding of HOX13 directly to the target *Hoxd* genes because these genes are fully transcribed by the C-DOM enhancers in the same distal cells. Therefore, we conclude that HOX13 proteins terminate T-DOM regulation by binding to this chromatin structure yet not necessarily to the silenced enhancer sequences, raising the possibility that the T-DOM is a global unit of regulation that could be switched on or off by factors not specific to any of the existing enhancer sequences.

The presence of HOXA13 bound at some of the activating enhancers at the *HoxA* locus (Berlivet et al. 2013) suggests that a similar positive effect may be at work. However, unlike *HoxD*, the *HoxA* cluster itself displayed at least three significant sites bound by HOXA13, in particular one covering the *Hoxa11* transcription unit, which is silent in distal cells, suggesting that HOX13 proteins may also have a direct effect on some *Hoxa* target genes. HOXA13 binding was also observed near *Hoxa7* and *Hoxa9*, two genes up-regulated in the distal limbs of *Hox13*^−/−^ mutants, suggesting that HOX13 proteins may help restrict the expression of these genes proximally by acting on local regulatory elements.

### The mechanism of TAD switching

**Figure 7.**
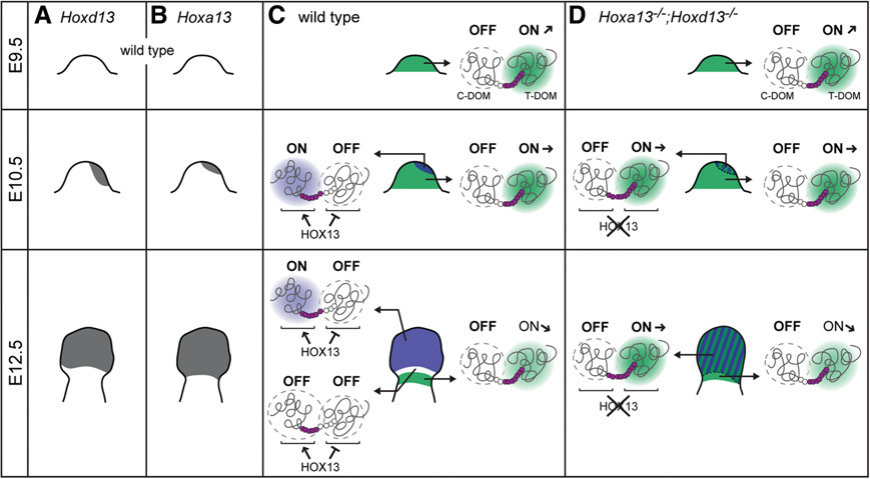
HOX13 function in the control of the regulatory switch at the *HoxD* locus. (*A*,*B*) Schematic *Hoxd13* (*A*) and *Hoxa13* (*B*) expression at E9.5, E10.5, and E12.5. Expression domains are in gray. (*C*) In the wild-type situation, the T-DOM operates in the early E9.5 limb bud. In E10.5 proximal limb cells, the T-DOM continues to drive *Hoxd* gene expression yet with a weaker activity. At this stage, distal cells (blue) individualize where *Hox13* genes start be transcribed. In these cells, HOX13 proteins both switch off the T-DOM and sustain strong transcription of *Hoxd13* by the C-DOM. In E12.5 limbs, proximal cells still weakly implement T-DOM regulation, while cells operating C-DOM regulation have expanded distally, likely in response to distal factors released by the AER. This expansion generates a zone of low Hox gene activity (white), which gives rise to the mesopodium (wrist and ankle). (*D*) In *Hoxa13*^−/−^*;Hoxd13*^−/−^ double-mutant limbs, the T-DOM normally operates in proximal cells at E9.5. Transcription of *Hoxd13* is initiated in distal cells at E10.5 (shaded blue cells), but, in the absence of HOX13 proteins, the C-DOM remains switched off, and the T-DOM continues to drive *Hoxd* genes in the most distal cells. The active C-DOM and T-DOM are shown by blue and green clouds, respectively. Active and inactive *Hoxd* genes are depicted with purple or white hexagons, respectively. The font size and arrows next to “ON” illustrate the various levels of T-DOM activity.

From this set of experiments, we propose a hypothesis for the bimodal *Hoxd* gene regulation during limb development. In the incipient bud, the T-DOM regulates the progressive transcriptional activity of *Hoxd8* to *Hoxd11*, which help organize and grow the proximal structures (Tarchini and Duboule 2006), whereas the C-DOM is inactive (Fig. 7C, top). At E10.5, along with distal growth and the release of secreted factors by the distal apical ectodermal ridge (AER), the *Shh* system and its feedback loop are fully implemented, partly under the control of HOX proteins present in the early bud (Knezevic et al. 1997; Tarchini et al. 2006; Zeller et al. 2009; Sheth et al. 2013). In turn, this signaling system triggers the activation of the C-DOM, leading to the appearance of *Hoxd13* transcripts in the most posterior and distal aspect of the bud (Fig. 7C, blue in the middle panel). The HOXD13 protein—along with HOXA13, also activated in the most distal cells—represses the activity of the T-DOM in distal cells, while, at the same time, likely in conjunction with AER factors, it sustains the positive activity of the C-DOM over *Hoxd13* to *Hoxd10* (Montavon et al. 2008). At E12.5, the domain of C-DOM activity has extended distally, where the concentrations of AER secreted factors are high, and some proximal cells where the C-DOM was initially active stop operating this regulation. In these presumptive cells of the mesopodium, both the T-DOM and the C-DOM are inactive, leading to only residual amounts of HOX protein. Cartilage models will fail to elongate and produce the wrist (Fig. 7B, bottom; Villavicencio-Lorini et al. 2010; Woltering and Duboule 2010; Gonzalez-Martin et al. 2014).

In the double *Hox13*^−/−^ mutant condition, the system initiates normally in the early bud, with the T-DOM in an “on” state (Fig. 7D, top), up to the start of *Hoxd13* transcription, which either does not occur or occurs at a very low level. In the absence of both HOX13 proteins, the T-DOM continues to operate in most distal cells, whereas the C-DOM remains inactive. In the absence of C-DOM function, T-DOM enhancers are even capable of activating *Hoxd13* transcription, although at a rather low level. The continuous action of T-DOM regulation produces a distal aspect of the limb bud lacking a clear “proximal” or “distal” identity (Fig. 7D, green and blue shaded areas), at least when considering our principal component analyses and the phenotype of such mutant fetuses. While they clearly show the presence of a smaller “digital plate” at an early stage, digits and more proximal structures are not subsequently formed (Fromental-Ramain et al. 1996). From this, we conclude that, while Hox genes are critical for the development of distal structures, they are not the sole genetic determinants of a “distal” limb identity.

The absence of a switch between TADs makes the appearance of HOX-negative cells impossible due to the nonrepression of T-DOM activity and concurrent nonsegregation of proximal and distal expression domains (Sheth et al. 2014). Consequently, such mutant limbs do not develop any clear mesopodial-like structures (Fromental-Ramain et al. 1996). In this view, the opposite functions of HOX13 proteins on the T-DOM and C-DOM appears as a parsimonious solution to make these regulations exclusive from one another, an essential prerequisite for the offset between proximal and distal expression domains and hence the development of the mesopodial articulation. While the molecular mechanisms underlying these antagonistic activities are unclear, they may involve various cofactors, as reported in other cases of transcription factors capable of such a dual activity (e.g., Tetreault et al. 2009).

### Evolution of *Hox* regulation in the transformation of paired appendages

During fin bud development, the transcript domains of *Hoxa13* and *Hoxd13* overlap with those of *Hoxa11* or *Hoxd11* (Sordino et al. 1995; van der Hoeven et al. 1996; Metscher et al. 2005; Ahn and Ho 2008), suggesting that the overall regulatory strategy is different and that fish lack structures homologous to tetrapod digits (see Woltering and Duboule 2010). However, a late distal phase of *Hox13* expression was detected in several fish species (Davis et al. 2007; Freitas et al. 2007; Johanson et al. 2007), leading to the proposal that the mammalian biphasic Hox regulation described above is an ancestral gnathostome character (Shubin et al. 2009). Our present work shows that distal expression for *Hox13* genes can be achieved without the proper digit regulatory controls, provided that the negative impact of HOX13 proteins on the system is abrogated, which is naturally the case in fish, as shown by their early coexpression with *Hox11* genes. In fact, the strict correlation proposed in tetrapods between the expression of *Hoxd13* and digit formation (Dolle et al. 1993) applies to only the protein and not the mRNAs because, in double *Hox13* mutant buds, *Hoxd13* transcripts can be located distally, resembling the wild-type situation yet in a domain that does not elicit digit formation. In this case, *Hoxd13* distal transcription is controlled by the “proximal system.”

In this context, one may hypothesize that either one or both ancestral fish TADs, which were observed at the *HoxD* locus (Woltering et al. 2014; Acemel et al. 2016), may have triggered rather general, proximal, and distal transcription of *Hoxd* genes in the budding fins (e.g., Gehrke et al. 2015) without any negative “cross-talk” between them. At some point during the evolution of tetrapods, the negative effect of HOX13 proteins over the T-DOM and their positive re-enforcement through the C-DOM appeared either via protein sequence modifications or through variations in the properties of these TADs. This step, along with the repression of *Hoxa11* by *Hoxa13*, leads to the two-phase, bimodal regulation described at the *HoxD* locus (Sheth et al. 2014; Woltering et al. 2014). Such a bimodal regulation allowed the self-enhanced transcription of *Hoxd13*, which, like *Hoxa13*, has the ability to stimulate growth and produce cartilage models and long bones of reduced size (Yokouchi et al. 1995a; Freitas et al. 2012), leading to digits. This also provided the mechanistic opportunity to offset the two domains of transcription and, as a consequence, produce the mesopodial articulation (Villavicencio-Lorini et al. 2010; Woltering and Duboule 2010; Andrey et al. 2013).

## Materials and methods

### Animal experimentation

All experiments were performed in agreement with the Swiss law on animal protection (LPA) under license number GE 81/14 (to D. Duboule). Chick embryos from a White Leghorn strain were incubated at 37.5°C and staged according to Hamburger and Hamilton (1992).

### In situ hybridization

Whole-mount in situ hybridizations were performed as described in Woltering et al. (2014).

### RNA extraction

Total RNA was extracted from individual pairs of wild-type or mutant proximal and distal forelimbs using the RNeasy microkit (Qiagen) following the manufacturer's instructions. A total of 100 ng of pure total RNA was amplified following standard Illumina procedure for polyA-selected RNA and sequenced on a HiSeq sequencer with a read length of 100 base pairs (bp). An in silico mutant version of the genome, including both the *Hoxd13/LacZ* and the *Hoxa13*/*Neomycin* alleles, was assembled, annotated, and used as the reference genome to map the *Hoxa13*^−/−^*;Hoxd13*^−/−^ transcriptomes with the mouse GCRm38 assembly. The mapping and FPKM (fragments per kilobase per million mapped fragments) calculations of expressed transcripts were performed using TopHat and Cufflinks implemented in a local Galaxy platform.

### 4C-seq

4C-seq was performed as described in Noordermeer et al. (2014). Pairs of proximal or distal forelimbs and hindlimbs were individually fixed with 2% formaldehyde, lysed, and stored at −80°C. After genotyping, pools of eight proximal or distal limbs were digested with NlaIII and DpnII as primary and secondary restriction enzymes, respectively, and ligation steps were performed using highly concentrated T4 DNA ligase (Promega, M1794). Inverse PCRs for amplification were carried out using primers for the *Hoxd11* and *Hoxd13* viewpoints (Noordermeer et al. 2011). PCR products were multiplexed and sequenced using a HiSeq sequencer from Illumina, and post-processing (demultiplexing, mapping, and 4C analysis) was conducted on the Bioinformatics and Biostatistics Core Facility HTSstation (http://htsstation.epfl.ch) (Noordermeer et al. 2011; David et al. 2014). Data were plotted on University of California at Santa Cruz (UCSC) genome bioinformatics site and smoothed with a window size of 11 fragments. A tentative relative quantification of the signal spanning both the *HoxD* telomeric and centromeric deserts was performed as described in Andrey et al. (2013). This quantification was not absolute and only reflected the balance of contacts between the two domains for each sample.

### Mutant and transgenic mice

Mice mutant for either the *Hoxa13* or the *Hoxd13* gene were those described in Fromental-Ramain et al. (1996) and Kondo et al. (1998). The mice carrying a deletion [*HoxD*^*Del(Nsi-Atf2)*^] or inversion [*HoxD*^*Inv(Nsi-Itga6)*^] of the C-DOM were described in Montavon et al. (2011) and Tschopp and Duboule (2011), respectively. To establish stable transgenic lines carrying either the CS39 or the CS65 enhancers, we generated a scaffold vector carrying the β-globin minimal promoter and the LacZ coding sequence (pSK-LacZ). The genomic region containing the regulatory elements CS39 (mm10, chromosome 2: 75,147,318–75,148,561) and CS65 (mm10, chromosome 2: 75,439,366–75,440,449) were PCR-amplified using specific primers and cloned into the pSK-LacZ vector. In both cases, the insert carrying the enhancer, β-globin minimal promoter, and LacZ coding sequence was excised from the vector backbone by digestion with KpnI–NotI. The fragment was gel-purified using the QIAquick gel extraction kit (Qiagen) and injected into fertilized oocytes. Three independent founders showing robust and reproducible LacZ expression in their offspring were selected for each construct. The presence of the transgene was assessed by PCR.

### ChIP-seq

Microdissected limb tissues from mouse and chick embryos were cross-linked with 1% formaldehyde/PBS for 18 min. Chromatin was sheared and used for each immunoprecipitation with antibodies against HOXA13 (Yokouchi et al. 1995a), H3K27ac (Abcam, ab4729), and H3K27me3 (Merck Millipore, 07-449), respectively. For ChIP-seq, at least 5 ng of purified DNA was used to make libraries according to the manufacturer's protocol (Illumina). The material was sequenced with 100-bp single-end reads on an Illumina HiSeq according to the manufacturer's specifications.

### Accession numbers

RNA-seq, ChIP-seq, and 4C-seq data sets are available from the NCBI Gene Expression Omnibus repository under accession number GSE79261. The control ChIP-seq data for H3K27ac in proximal limb can be found under GSM1104588. Any other information for HOXA13 ChIP-seq data and ChIP-seq data from *Hoxa13*^−/−^*;Hoxd13*^−/−^ embryos is available on request.

## Supplementary Material

Supplemental Material
