# Supplementary material for: A role for HOX13 proteins in the regulatory switch between TADs at the HoxD locus
Source: Genes Dev. 2016 May 15;30(10):1172–86. doi: 10.1101/gad.281055.116 (PMC4888838; doi:10.1101/gad.281055.116)
Supplement: Supplemental Material [file supp_30_10_1172__index.html]

A role for HOX13 proteins in the regulatory switch between TADs at the HoxD locus — A role for HOX13 proteins in the regulatory switch between TADs at the HoxD locus — Supplemental Material 

# A role for HOX13 proteins in the regulatory switch between TADs at the *HoxD* locus

## Supplemental Material

- Supplemental\_material\_index.pdf
- Supplemental\_methods.pdf
- Supplementary\_references.pdf
- Supplemental\_Fig\_S1.pdf
- Supplemental\_Fig\_S2.pdf
- Supplemental\_Fig\_S3.pdf
- Supplemental\_Fig\_S4.pdf
- Supplemental\_Fig\_S5.pdf
- Supplemental\_Fig\_S6.pdf
- Supplemental\_Fig\_S7.pdf
