# Supplementary material for: A role for HOX13 proteins in the regulatory switch between TADs at the HoxD locus
Source: Genes Dev. 2016 May 15;30(10):1172–86. doi: 10.1101/gad.281055.116 (PMC4888838; doi:10.1101/gad.281055.116)
Supplement: Supplemental Material [file supp_gad.281055.116_Supplemental_material_index.pdf]

HOX13 PROTEINS CONTROL THE REGULATORY SWITCH BETWEEN TADs AT THE *HoxD* LOCUS

SUPPLEMENTARY MATERIAL

Leonardo Beccari[1,#], Nayuta Yakushiji-Kaminatsui[2,#], Joost M. Woltering[1,a,#], Anamaria Necsulea[2], Nicolas Lonfat[2,b], Edgardo Rodriguez-Carballo[1], Benedicte Mascrez[1], Shiori Yamamoto[3], Atsushi Kuroiwa[3] and Denis Duboule[1,2,*]

[1]Department of Genetics and Evolution, University of Geneva, 1211 Geneva 4, Switzerland, [2]School of Life Sciences, Federal Institute of Technology, Lausanne, 1015 Lausanne, Switzerland, [3]Division of Biological Science, Graduate School of Science, Nagoya University, Furo-cho, Chikusa-ku, Nagoya, Japan.

[a]Present address: Department of Biology, University of Konstanz, Universitätsstraße 10, 78457, Konstanz, Germany

[b]Present address: Department of Genetics and Department of Ophthalmology, Harvard Medical School, Boston, MA 02115, USA.

[#]These authors equally contributed to this work

CONTENT

***Extended Materials and Methods**

***Supplementary Figures S1 (related to Figure 1)**

***Supplementary Figures S2 (related to Figure 1)**

***Supplementary Figures S3 (related to Figure 3)**

***Supplementary Figures S4 (related to Figure 3)**

***Supplementary Figures S5 (related to Figure 4)**

***Supplementary Figures S6 (related to Figure 4)**

***Supplementary Figures S7 (related to Figure 5)**

***References**
