# Supplementary material for: A role for HOX13 proteins in the regulatory switch between TADs at the HoxD locus
Source: Genes Dev. 2016 May 15;30(10):1172–86. doi: 10.1101/gad.281055.116 (PMC4888838; doi:10.1101/gad.281055.116)
Supplement: Supplemental Material [file supp_gad.281055.116_Supplemental_methods.pdf]

EXTENDED MATERIALS AND METHODS

Animal experimentation. All experiments were performed in agreement with the Swiss law on animal protection (LPA), under license No GE 81/14 (to DD).

***In situ* hybridization.** Whole mount *in situ* hybridizations were performed as described in (Woltering et al., 2014). Probes for the *Hoxa11, Hoxa13, Hoxd8, Hoxd10, Hoxd12, Hoxd13* and *Evx2* genes were synthetized and purified as previously reported (Herault et al., 1996; Woltering et al., 2014). Plasmids encoding the cDNAs of the *Prrx2* and *Dbx2* genes were purchased from Addgene and probes were synthetized as previously reported (Pierani et al., 1999; Stelnicki et al., 1998). Right or left forelimbs were dissected from stained embryos and photographied dorsally with a Leica MZ16 stereomicroscope. Pictures from left forelimbs are displayed inverted.

RNA extraction. Total RNA was extracted from individual pairs of either wild type or double *Hox13*$^{-/-}$ mutant proximal and distal forelimb buds, using the RNeasy Micro Kit (Qiagen) following manufacturer instructions. A total of 100ng of pure total RNA was amplified following standard Illumina procedure for polyA-selected RNA.

RNA-seq data generation. RNA sequencing (RNA-seq) libraries were prepared with the Illumina TruSeq Stranded mRNA protocol and sequenced on a HiSeq 2500 machine, as single-end, 100 base pairs (bp) reads. The preparation of libraries and sequencing were performed by the genomic platform of the University of Geneva.

RNA-seq data analysis. A mutant version of the genome, encoding the *Hoxd13*/*LacZ* and the *Hoxa13*/*Neo*$^{+}$ alleles (Fromental-Ramain et al., 1996; Kondo et al., 1998), was assembled and annotated and used as reference genome to map the *Hoxa13*$^{-/-}$ and *Hoxd13*$^{-/-}$ RNA-seq data. The mutant genome was constructed from the mouse GRCm38/mm10 assembly. The RNA-seq data were aligned against their respective genomes using TopHat release 2.0.9 (Kim et al., 2013). Gene expression levels were computed based on Ensembl release 82 annotations (Yates et al., 2016), using Cufflinks release 2.2.1 (Trapnell et al., 2010). Ensembl annotations were filtered to exclude potentially non-functional transcripts associated with protein-coding genes (e.g., transcripts without protein-coding potential, including retained introns or targeted by the nonsense-mediated decay mechanism); all transcripts were kept for non-coding genes. Gene expression was evaluated using the FPKM (Fragments per Kilobase of exon per Million mapped reads) measure, computed based on all aligned reads and the multiple mapping correction procedure implemented in Cufflinks. TopHat and Cufflinks were implemented

using a local Galaxy server (Blankenberg et al., 2010). TopHat output files were processed with SAMTools (Li et al., 2009) and BedTools (Quinlan, 2014) to extract the unique read coverage along the genome. For visualisation and comparison across samples, the read coverage was divided by the number of millions of mapped reads in each sample. The principal component analysis (PCA) was performed using the ade4 package (Dray and Dufour, 2007) in R. Prior to PCA analysis, FPKM levels were log2-transformed, after adding an offset of 1 to each value. The log2-transformed values were then centred across samples to obtain an average of 0 for each gene; no variance scaling was performed.

4C-seq. Chromosome conformation capture (4C-seq) was performed as described in (Noordermeer et al., 2014). Pairs of proximal or distal fore- and hindlimbs were individually fixed with 2% formaldehyde, lysed and stored at -80°C. After genotyping, pools of eight proximal or distal limbs were digested with *NlaIII* and *DpnII* as primary and secondary restriction enzymes, respectively, and ligation steps were performed using high concentrated T4 DNA ligase (M1794, Promega). Inverse PCRs for amplification were carried out using primers for the *Hoxd11* and *Hoxd13* viewpoints (Noordermeer et al., 2011). PCR products were multiplexed and sequenced using a HiSeq sequencer from Illumina and post-processing (de-multiplexing, mapping and 4C analysis) was conducted on the BBCF HTSstation (http://htsstation.epfl.ch) (David et al., 2014; Noordermeer et al., 2011). Data were plotted on UCSC and smoothed with a window size of 11 fragments. A tentative relative quantification of the signal spanning both the *HoxD* telomeric or centromeric deserts was performed as described in (Andrey et al., 2013). This quantification is not absolute and only reflects the balance of contacts between the two C-DOM and T-DOM domains for each sample. For visualization, the scale for each sample and viewpoint was normalized according to both the total number of mapped reads and the enrichment of the viewpoint.

Mutant and transgenic mice. Mice mutant for either the *Hoxa13* or the *Hoxd13* gene were those described in (Fromental-Ramain et al., 1996; Kondo et al., 1998). To establish stable transgenic lines carrying either the CS39 or the CS65 enhancers, we generated a scaffold vector carrying the beta-globin minimal promoter and the LacZ coding sequence (pSk-LacZ). The genomic region containing the regulatory elements CS39 (chr2: 75,147,318-75,148,561) and CS65 (chr2: 75,439,366-75,440,449) were PCR amplified using specific primers (CS39F: ACAGTGCAGGTAGTAGAGAG, CS39R: GTTCTTACATTTTGAGATC; CS65F: CATCTGATCCCAGTGAGGAC, CS65R: ACATGTGATCCAGGGAACTG) and cloned into the pSK-LacZ vector. In both cases, the insert carrying the enhancer, b-globin minimal

promoter and the LacZ coding sequence was excised from the vector backbone by digestion with KpnI-NotI. The fragment was gel-purified using the QIAquick Gel Extraction Kit (QIAGEN) and injected into fertilized oocytes. Three independent founders showing robust and reproducible LacZ expression in their offspring were selected for each construct. The presence of the transgene was assessed by PCR.

ChIP-seq. Micro-dissected proximal and distal segments of E12.5 limbs either from wild type CD1 mice, or from various combinations of *Hoxa13;Hoxd13* mutant alleles, and of stage HH28 (Hamburger and Hamilton, 1992) forelimbs from chick were used for ChIP-seq. Samples were fixed in 1% formaldehyde/PBS for 18 minutes at room temperature, washed at least three times with cold PBS containing protease inhibitor and stored at -80°C. Pools of ten pairs of wild type proximal or distal forelimbs, or of three pairs of *Hoxa13*$^{-/-}$;*Hoxd13*$^{-/-}$ double mutant proximal or distal fore- and hindlimbs, or thirteen pairs of chick proximal or distal forelimbs were used for each experiment. The samples were treated with lysis buffer (1% SDS, 10 mM EDTA, 50 mM Tris-HCl and protease inhibitor) for 10 min on ice, followed by RIPA buffer (TE with 140 mM NaCl, 0.1% deoxycholate, 0.1% SDS, 1% TritonX-100 and protease inhibitor) and fragmented to a range of 200-500 bp by a Biorupter sonication device (Diagenode). Pierce™ Protein A/G magnetic beads (88803, Thermo Scientific) were conjugated with each antibody in blocking buffer (PBS, 0.5% Tween20, 0.5% BSA and protease inhibitor) for 4 hrs at 4°C and washed with blocking buffer. Chromatin was incubated with the prepared protein A/G beads overnight at 4°C with rotation. 4 μg of anti-HOXA13 (Yokouchi et al., 1995), 3 μg of H3K27ac (ab4729, Abcam) and 3 μg of H3K27me3 (07-449, Millipore) were used for each immunoprecipitation. Samples were washed 6 times with RIPA buffer, twice with RIPA/500 mM NaCl buffer, twice with LiCl wash buffer (TE, 250 mM LiCl, 0.5% NP-40 and 0.5% deoxycholate) and twice with TE, followed by elution in (10 mM Tris-HCl, 5 mM EDTA, 300 mM NaCl and 0.1% SDS). Cross-links were reversed overnight at 65°C and ChIPed DNA was treated with RNase A and proteinase K, and purified by phenol chloroform extraction. For ChIP-seq, at least 5 ng of purified DNA were used to make libraries according to the manufacturer's protocol (Illumina). The material was sequenced with 100 bp single-end reads on the Illumine HiSeq according to the manufacturer's specifications. Demultiplexed ChIP-seq reads were mapped onto the Genome Reference Consortium GRCm38 (mm10) or onto the International Chicken Genome Reference Consortium Gallus_gallus-4.0 (galGal4), using Bowtie (Galaxy Tool Version 1.1.2) (Langmead et al., 2009), with parameters "-m1 –strata –best" according to the

conditions described previously (Riising et al., 2014). Both unmapped regions containing adaptors and contamination and PCR duplicates were removed from mapped reads. Model-Based Analysis of ChIP-seq (MACS) (Galaxy version 1.0.1) (Zhang et al., 2008) was used to identify HOXA13 peak regions using the input as control, with parameters of *p*-value cutoff of 10^{-7} and at least 7-fold enrichment. These analyses were performed on the public server of the Galaxy Project (https://usegalaxy.org/)(Hillman-Jackson et al., 2012). By using bamCompare (Galaxy Tool Version 1.5.9.1.0) from the Galaxy deepTools webserver (http://deeptools.ie-freiburg.mpg.de/), ChIP and input data were normalized and compared to compute the difference of the number of reads or log2 of the number of reads ratio (Ramirez et al., 2014).
