# Supplementary material for: A role for HOX13 proteins in the regulatory switch between TADs at the HoxD locus
Source: Genes Dev. 2016 May 15;30(10):1172–86. doi: 10.1101/gad.281055.116 (PMC4888838; doi:10.1101/gad.281055.116)
Supplement: Supplemental Material [file supp_gad.281055.116_Supplementary_references.pdf]

REFERENCES

Andrey, G., Montavon, T., Mascrez, B., Gonzalez, F., Noordermeer, D., Leleu, M., Trono, D., Spitz, F., and Duboule, D. (2013). A switch between topological domains underlies HoxD genes collinearity in mouse limbs. Science *340*, 1234167.

Berlivet, S., Paquette, D., Dumouchel, A., Langlais, D., Dostie, J., and Kmita, M. (2013). Clustering of tissue-specific sub-TADs accompanies the regulation of HoxA genes in developing limbs. Plos Genetics *9*, e1004018.

Blankenberg, D., Von Kuster, G., Coraor, N., Ananda, G., Lazarus, R., Mangan, M., Nekrutenko, A., and Taylor, J. (2010). Galaxy: a web-based genome analysis tool for experimentalists. Current protocols in molecular biology / edited by Frederick M Ausubel [et al] *Chapter 19*, Unit 19 10 11-21.

David, F.P., Delafontaine, J., Carat, S., Ross, F.J., Lefebvre, G., Jarosz, Y., Sinclair, L., Noordermeer, D., Rougemont, J., and Leleu, M. (2014). HTSstation: A Web Application and Open-Access Libraries for High-Throughput Sequencing Data Analysis. PLoS ONE *9*, e85879.

Dray, S., and Dufour, A.B. (2007). The ade4 package: Implementing the duality diagram for ecologists. J Stat Softw *22*, 1-20.

Fromental-Ramain, C., Warot, X., Messadecq, N., LeMeur, M., Dolle, P., and Chambon, P. (1996). Hoxa-13 and Hoxd-13 play a crucial role in the patterning of the limb autopod. Development *122*, 2997-3011.

Hamburger, V., and Hamilton, H.L. (1992). A series of normal stages in the development of the chick embryo. 1951. Dev Dyn *195*, 231-272.

Herault, Y., Hraba-Renevey, S., van der Hoeven, F., and Duboule, D. (1996). Function of the Evx-2 gene in the morphogenesis of vertebrate limbs. Embo J *15*, 6727-6738.

Hillman-Jackson, J., Clements, D., Blankenberg, D., Taylor, J., Nekrutenko, A., and Galaxy, T. (2012). Using Galaxy to perform large-scale interactive data analyses. Current protocols in bioinformatics / editoral board, Andreas D Baxevanis [et al] *Chapter 10*, Unit10 15.

Hsieh-Li, H.M., Witte, D.P., Weinstein, M., Branford, W., Li, H., Small, K., and Potter, S.S. (1995). Hoxa 11 structure, extensive antisense transcription, and function in male and female fertility. Development *121*, 1373-1385.

Kim, D., Pertea, G., Trapnell, C., Pimentel, H., Kelley, R., and Salzberg, S.L. (2013). TopHat2: accurate alignment of transcriptomes in the presence of insertions, deletions and gene fusions. Genome Biol *14*, R36.

Kondo, T., Zákány, J., and Duboule, D. (1998). Control of colinearity in AbdB genes of the mouse HoxD complex. Molecular Cell *1*, 289-300.

Langmead, B., Trapnell, C., Pop, M., and Salzberg, S.L. (2009). Ultrafast and memory-efficient alignment of short DNA sequences to the human genome. Genome Biol *10*, R25.

Li, H., Handsaker, B., Wysoker, A., Fennell, T., Ruan, J., Homer, N., Marth, G., Abecasis, G., Durbin, R., and Genome Project Data Processing, S. (2009). The Sequence Alignment/Map format and SAMtools. Bioinformatics *25*, 2078-2079.

Lonfat, N., Montavon, T., Darbellay, F., Gitto, S., and Duboule, D. (2014). Convergent evolution of complex regulatory landscapes and pleiotropy at Hox loci. Science *346*, 1004-1006.

Noordermeer, D., Leleu, M., Schorderet, P., Joye, E., Chabaud, F., and Duboule, D. (2014). Temporal dynamics and developmental memory of 3D chromatin architecture at Hox gene loci. eLife *3*, e02557.

Noordermeer, D., Leleu, M., Splinter, E., Rougemont, J., De Laat, W., and Duboule, D. (2011). The dynamic architecture of Hox gene clusters. Science *334*, 222-225.

Pierani, A., Brenner-Morton, S., Chiang, C., and Jessell, T.M. (1999). A sonic hedgehog-independent, retinoid-activated pathway of neurogenesis in the ventral spinal cord. Cell *97*, 903-915.

Quinlan, A.R. (2014). BEDTools: The Swiss-Army Tool for Genome Feature Analysis. Current protocols in bioinformatics / editoral board, Andreas D Baxevanis [et al] *47*, 11 12 11-11 12 34.

Ramirez, F., Dundar, F., Diehl, S., Gruning, B.A., and Manke, T. (2014). deepTools: a flexible platform for exploring deep-sequencing data. Nucleic Acids Res *42*, W187-191.

Riising, E.M., Comet, I., Leblanc, B., Wu, X., Johansen, J.V., and Helin, K. (2014). Gene silencing triggers polycomb repressive complex 2 recruitment to CpG islands genome wide. Mol Cell *55*, 347-360.

Stelnicki, E.J., Arbeit, J., Cass, D.L., Saner, C., Harrison, M., and Largman, C. (1998). Modulation of the human homeobox genes PRX-2 and HOXB13 in scarless fetal wounds. The Journal of investigative dermatology *111*, 57-63.

Trapnell, C., Williams, B.A., Pertea, G., Mortazavi, A., Kwan, G., van Baren, M.J., Salzberg, S.L., Wold, B.J., and Pachter, L. (2010). Transcript assembly and quantification by RNA-Seq reveals unannotated transcripts and isoform switching during cell differentiation. Nat Biotechnol *28*, 511-515.

Wang, K.C., Yang, Y.W., Liu, B., Sanyal, A., Corces-Zimmerman, R., Chen, Y., Lajoie, B.R., Protacio, A., Flynn, R.A., Gupta, R.A., *et al.* (2011). A long noncoding RNA maintains active chromatin to coordinate homeotic gene expression. Nature *472*, 120-124.

Woltering, J.M., Noordermeer, D., Leleu, M., and Duboule, D. (2014). Conservation and divergence of regulatory strategies at Hox Loci and the origin of tetrapod digits. PLoS Biol *12*, e1001773.

Yates, A., Akanni, W., Amode, M.R., Barrell, D., Billis, K., Carvalho-Silva, D., Cummins, C., Clapham, P., Fitzgerald, S., Gil, L., *et al.* (2016). Ensembl 2016. Nucleic Acids Res *44*, D710-716.

Yokouchi, Y., Nakazato, S., Yamamoto, M., Goto, Y., Kameda, T., Iba, H., and Kuroiwa, A. (1995). Misexpression of Hoxa-13 induces cartilage homeotic transformation and changes cell adhesiveness in chick limb buds. Genes Dev *9*, 2509-2522.

Zhang, Y., Liu, T., Meyer, C.A., Eeckhoute, J., Johnson, D.S., Bernstein, B.E., Nusbaum, C., Myers, R.M., Brown, M., Li, W., *et al.* (2008). Model-based analysis of ChIP-Seq (MACS). Genome Biol *9*, R137.
