# Supplementary material for: A role for HOX13 proteins in the regulatory switch between TADs at the HoxD locus
Source: Genes Dev. 2016 May 15;30(10):1172–86. doi: 10.1101/gad.281055.116 (PMC4888838; doi:10.1101/gad.281055.116)
Supplement: Supplemental Material [file supp_gad.281055.116_Supplemental_Fig_S1.pdf]

Supplemental Figure 1 (Figure S1, related to Figure 1)

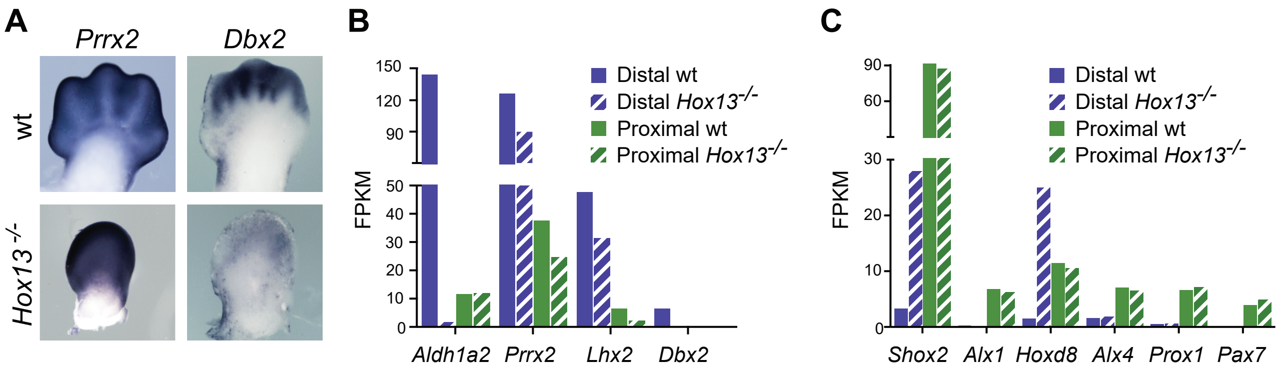

Figure S1. Expression of distal genes in *Hoxa13*$^{-/-}$*;Hoxd13*$^{-/-}$ double mutant forelimbs. (A) *In situ* hybridization analysis showing the expression of *Prrx2* and *Dbx2* (top) in the forelimbs of either wild type (wt) or *Hoxa13*$^{-/-}$*;Hoxd13*$^{-/-}$ (*Hox13*$^{-/-}$) double mutant specimen at E12.5 to E13. **(B-C)** Bar graph showing the normalized reads count expressed as fragments per kilobase per million (FPKM) of mapped reads of different genes in the proximal and distal limb of wt or *Hox13*$^{-/-}$ mutant embryos. Some of the genes normally enriched in the control distal limb were strongly reduced in *Hox13*$^{-/-}$ mutants while others were not significantly affected. Likewise, only a subset of proximally enriched genes were up-regulated in control distal forelimb.
