# Supplementary material for: A role for HOX13 proteins in the regulatory switch between TADs at the HoxD locus
Source: Genes Dev. 2016 May 15;30(10):1172–86. doi: 10.1101/gad.281055.116 (PMC4888838; doi:10.1101/gad.281055.116)
Supplement: Supplemental Material [file supp_gad.281055.116_Supplemental_Fig_S2.pdf]

Supplemental Figure 2 (Figure S2, related to Figure 1)

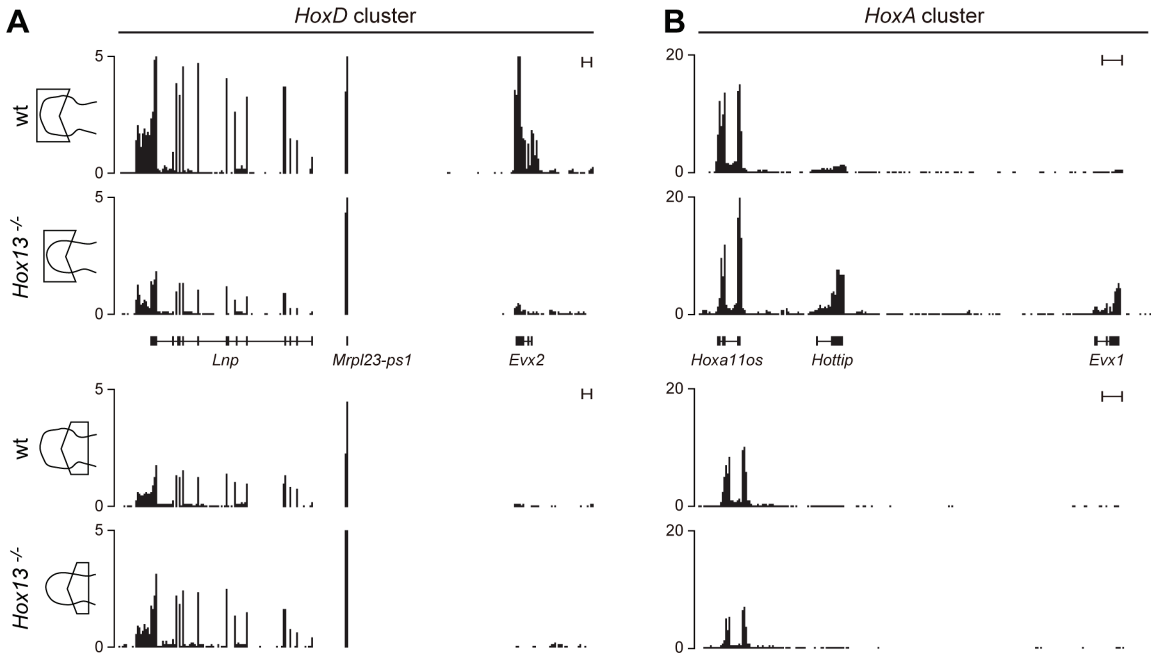

Figure S2. Expression of non-*Hox* genes at both the *HoxD* and *HoxA* loci in double *Hoxa13*$^{-/-}$*;Hoxd13*$^{-/-}$ forelimb buds. (A-B) Transcription profiles of the *Hox* antisense strands in the distal and proximal domains of either wild type (wt) or *Hoxa13*$^{-/-}$*;Hoxd13*$^{-/-}$ (*Hox13*$^{-/-}$) double mutants forelimbs at both the *HoxD* (left) and *HoxA* (right) loci. The Y axis represents the strand-specific RNA-seq read counts, divided by the total number of million mapped reads. Transcriptomes from *Hox13*$^{-/-}$ mutant were aligned to a manually engineered version of the genome including the sequences introduced in the targeted approaches (see Material and Methods). For visualization, the profiles of the wild type *Hoxa* and *Hoxd* genes lying downstream of the *Neomycin* (*Neo*) and the *LacZ* (*Lac*) integration sites were shifted to align with the mutant genome annotations. Scale bar, 4 kb. Both the *Lnp* and *Evx2* genes were down-regulated in *Hox13*$^{-/-}$ mutant limbs. At the *HoxA* locus, while the *Hotip* LncRNA (Wang et al., 2011) and the *Evx1* gene were also down regulated in mutant limb buds, the *Hoxa11* antisense transcript (Hsieh-Li et al., 1995) remained unaffected.
