# Supplementary material for: A role for HOX13 proteins in the regulatory switch between TADs at the HoxD locus
Source: Genes Dev. 2016 May 15;30(10):1172–86. doi: 10.1101/gad.281055.116 (PMC4888838; doi:10.1101/gad.281055.116)
Supplement: Supplemental Material [file supp_gad.281055.116_Supplemental_Fig_S3.pdf]

Supplemental Figure 3 (Figure S3, related to Figure 3)

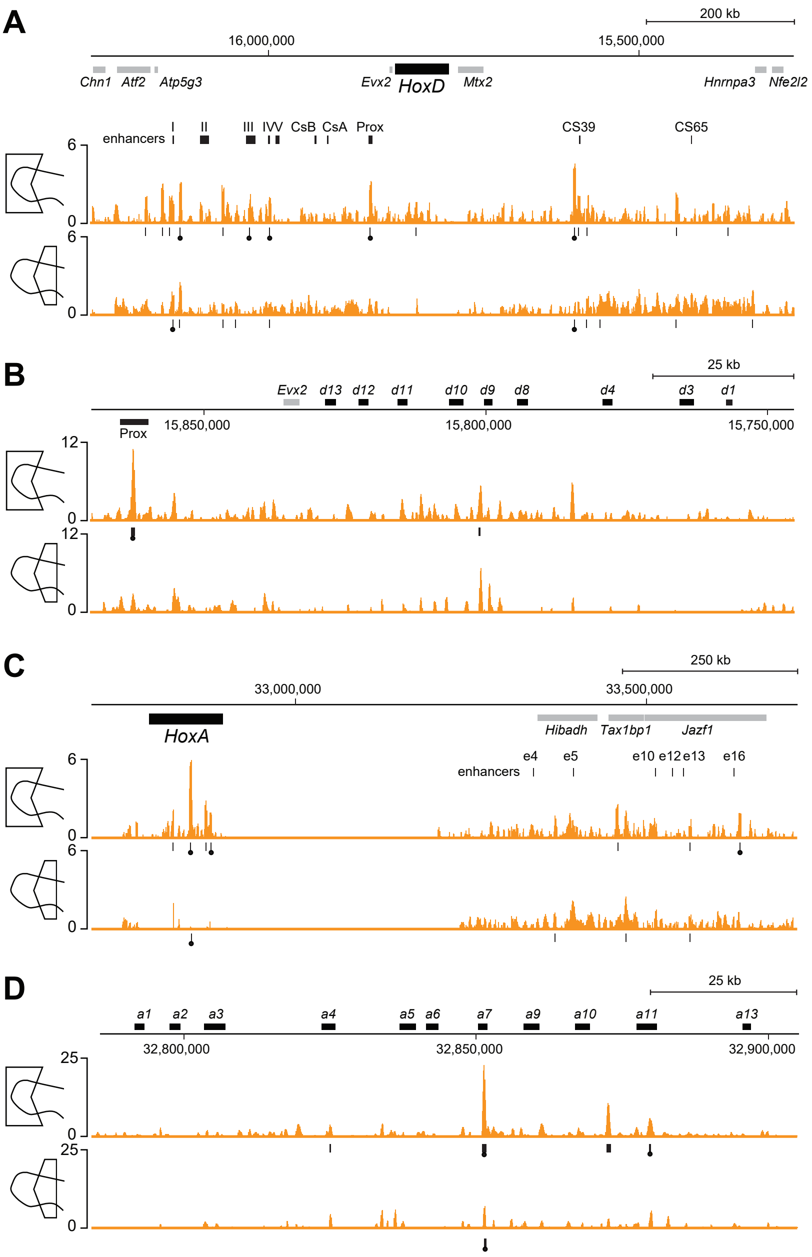

Figure S3. HOXA13 ChIP-seq profile in distal and proximal domains of chick forelimbs at stage HH28. Enrichment (y-axis) is shown as the difference of the normalized number of reads between ChIPed and input material. In each case, the upper and lower track shows the dissected distal and proximal regions of the forelimb, respectively. *HoxA* and *HoxD* regulatory elements conserved between mouse and chicken were identified using the LiftOver function of the UCSC genome browser and depicted as black rectangle above the binding profiles in tracks **A**, **B** and **C**. HOXA13 peaks as detected by MACS (Zhang et al., 2008) and common between mouse and chick samples are marked with a circle (see also in Figure 3). **(A)** View of the *HoxD* regulatory landscapes and **(B)** enlargement of the *HoxD* cluster. While the strongest signals match previously defined regulatory islands, some perhaps significant yet weaker peaks are scored over the *Hoxd9* locus. **(C)** View of a 1 Mb large region including the *HoxA* cluster and its digit enhancers where HOXA13 binding is enriched. **(D)** A zoom in the *HoxA* cluster show binding specificity comparable to that scored in the mouse counterpart.
