# Supplementary material for: A role for HOX13 proteins in the regulatory switch between TADs at the HoxD locus
Source: Genes Dev. 2016 May 15;30(10):1172–86. doi: 10.1101/gad.281055.116 (PMC4888838; doi:10.1101/gad.281055.116)
Supplement: Supplemental Material [file supp_gad.281055.116_Supplemental_Fig_S4.pdf]

Supplemental Figure 4 (Figure S4, related to Figure 3)

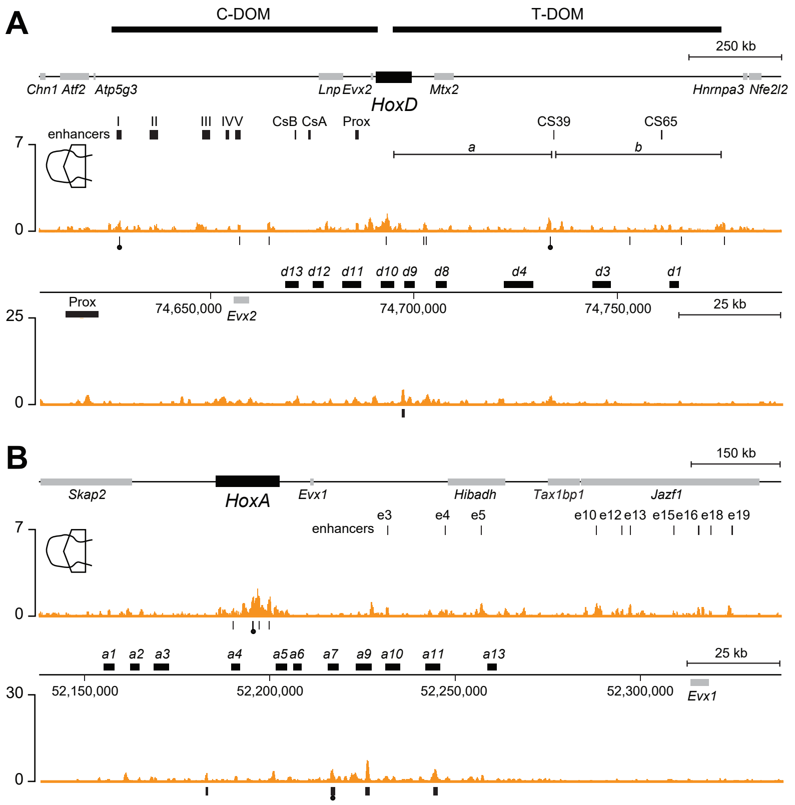

Figure S4. HOXA13 ChIP-seq profile at both *HoxD* and *HoxA* loci in control proximal forelimbs at E12.5. Enrichment (y-axis) is shown as the difference of the normalized number of reads between ChIP and input samples. Common Peaks of bound HOXA13 as determined by MACS (Zhang et al., 2008) and common between both mouse and chick limb samples are marked with a circle (see also Figure 3 and Figure S3). Bracketed regions '*a*' and '*b*' represent the two sub TADs of T-DOM (as in Figure 4). **(A)** Analysis of the *HoxD* regulatory landscapes (top) revealed some weak signals (e.g. island I or CS39, indicated on top), likely derived from a light contamination in dissections between distal and proximal samples. Close-up view the *HoxD* cluster with close-to undetectable signals. **(B)** View of the *HoxA* cluster (bottom) and its limb-enhancers containing region, indicated on top (Berlivet et al., 2013). As for **(A)**, the peaks detected both on enhancer sequences and over the *HoxA* cluster, correspond to those scored in the distal sample, likely revealing a contamination.
