# Supplementary material for: A role for HOX13 proteins in the regulatory switch between TADs at the HoxD locus
Source: Genes Dev. 2016 May 15;30(10):1172–86. doi: 10.1101/gad.281055.116 (PMC4888838; doi:10.1101/gad.281055.116)
Supplement: Supplemental Material [file supp_gad.281055.116_Supplemental_Fig_S5.pdf]

Supplemental Figure 5 (Figure S5, related to Figure 4)

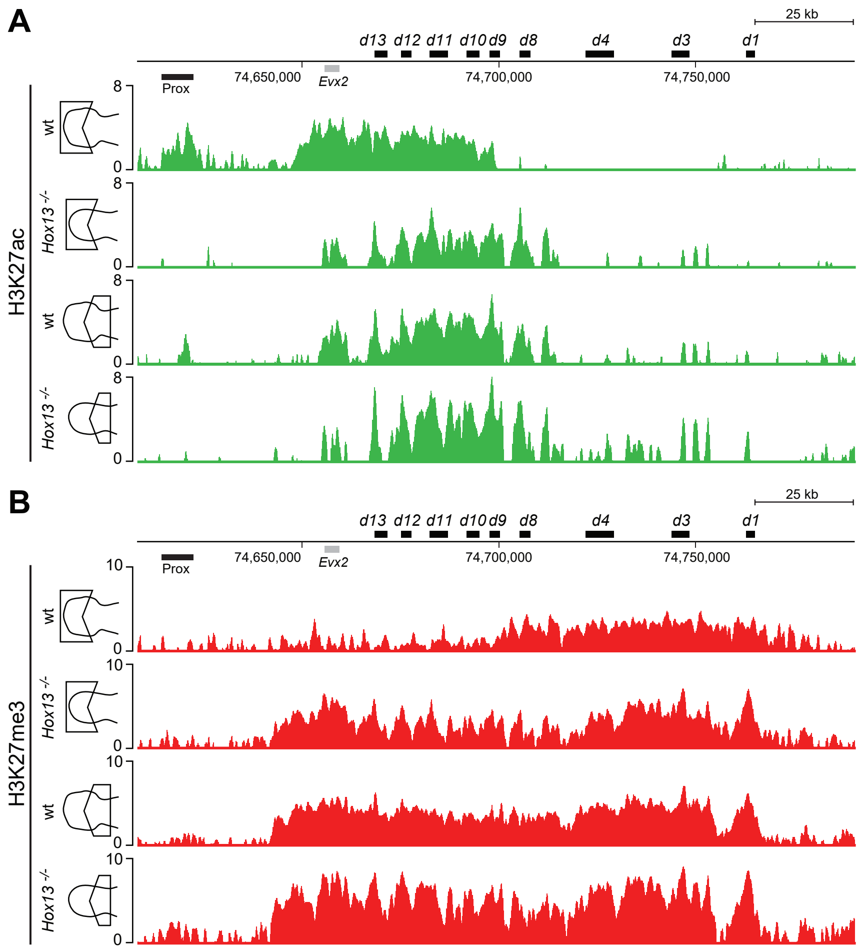

Figure S5. ChIP-seq profiles of H3K27ac and H3K27me3 in E12.5 distal and proximal of limbs. (A) H3K27ac marks at the *HoxD* cluster in both wild type (wt) and *Hoxa13*$^{-/-}$;*Hoxd13*$^{-/-}$ (*Hox13*$^{-/-}$) mutant distal or proximal forelimb buds. The profile in the mutant distal sample (track 2) resembles that of the control proximal sample (track 3). The digit enhancer Prox is heavily decorated in control distal cells (track 1), whereas totally devoid of H3K27ac marks in the mutant counterpart (track 2). **(B)** H3K27me3 profiles at the *HoxD* cluster in wild type and *Hox13*$^{-/-}$ mutant distal or proximal forelimb buds. Profiles complementary to those under **(A)** were scored, with a strong coverage over *Hoxd13* and *Hoxd12* in mutant distal cells, the latter genes being no longer activated by C-DOM, yet slightly transcribed by the T-DOM enhancers. Enrichment (y-axis) is shown as the log2 ratio of the normalized number of reads between ChIPed and input samples.
