# Supplementary material for: A role for HOX13 proteins in the regulatory switch between TADs at the HoxD locus
Source: Genes Dev. 2016 May 15;30(10):1172–86. doi: 10.1101/gad.281055.116 (PMC4888838; doi:10.1101/gad.281055.116)
Supplement: Supplemental Material [file supp_gad.281055.116_Supplemental_Fig_S6.pdf]

Supplemental Figure 6 (Figure S6, related to Figure 4)

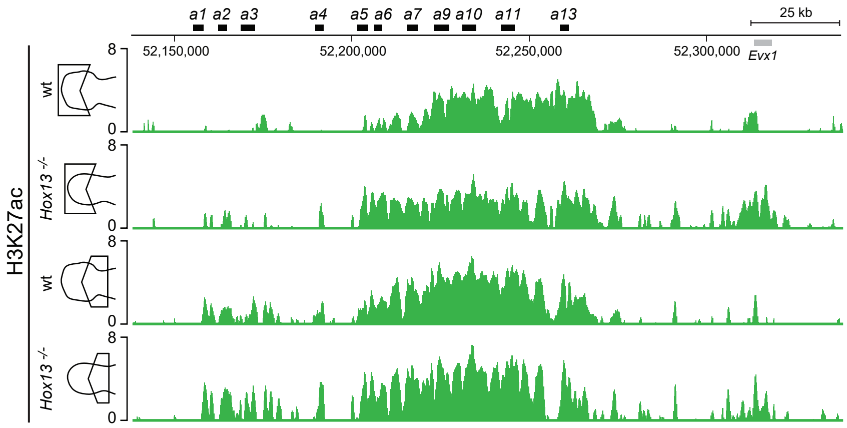

Figure S6. Profiles of H3K27ac marks over the *HoxA* cluster in both wild type and *Hoxa13*$^{-/-}$*;Hoxd13*$^{-/-}$ double mutant distal or proximal E12.5 forelimbs. H3K27ac ChIP-seq data at *HoxA* cluster in the wild type distal and proximal forelimb or *Hoxa13*$^{-/-}$*;Hoxd13*$^{-/-}$ (*Hox13*$^{-/-}$) mutant distal and proximal limb. The profiles obtained with the mutant distal samples resemble those derived from the control proximal samples. Note the decrease, in mutant distal cells, in the level of H3K27 acetylation over the *Hoxa11* to *Hoxa13* loci, as well as the increase of such marks over the *Hoxa1* to *Hoxa7* region, when compared to control. Enrichment (y-axis) is shown as the log2 ratio of the normalized number of reads between ChIPed and input material.
