# Supplementary material for: A role for HOX13 proteins in the regulatory switch between TADs at the HoxD locus
Source: Genes Dev. 2016 May 15;30(10):1172–86. doi: 10.1101/gad.281055.116 (PMC4888838; doi:10.1101/gad.281055.116)
Supplement: Supplemental Material [file supp_gad.281055.116_Supplemental_Fig_S7.pdf]

Supplemental Figure 7 (Figure S7, related to Figure 5)

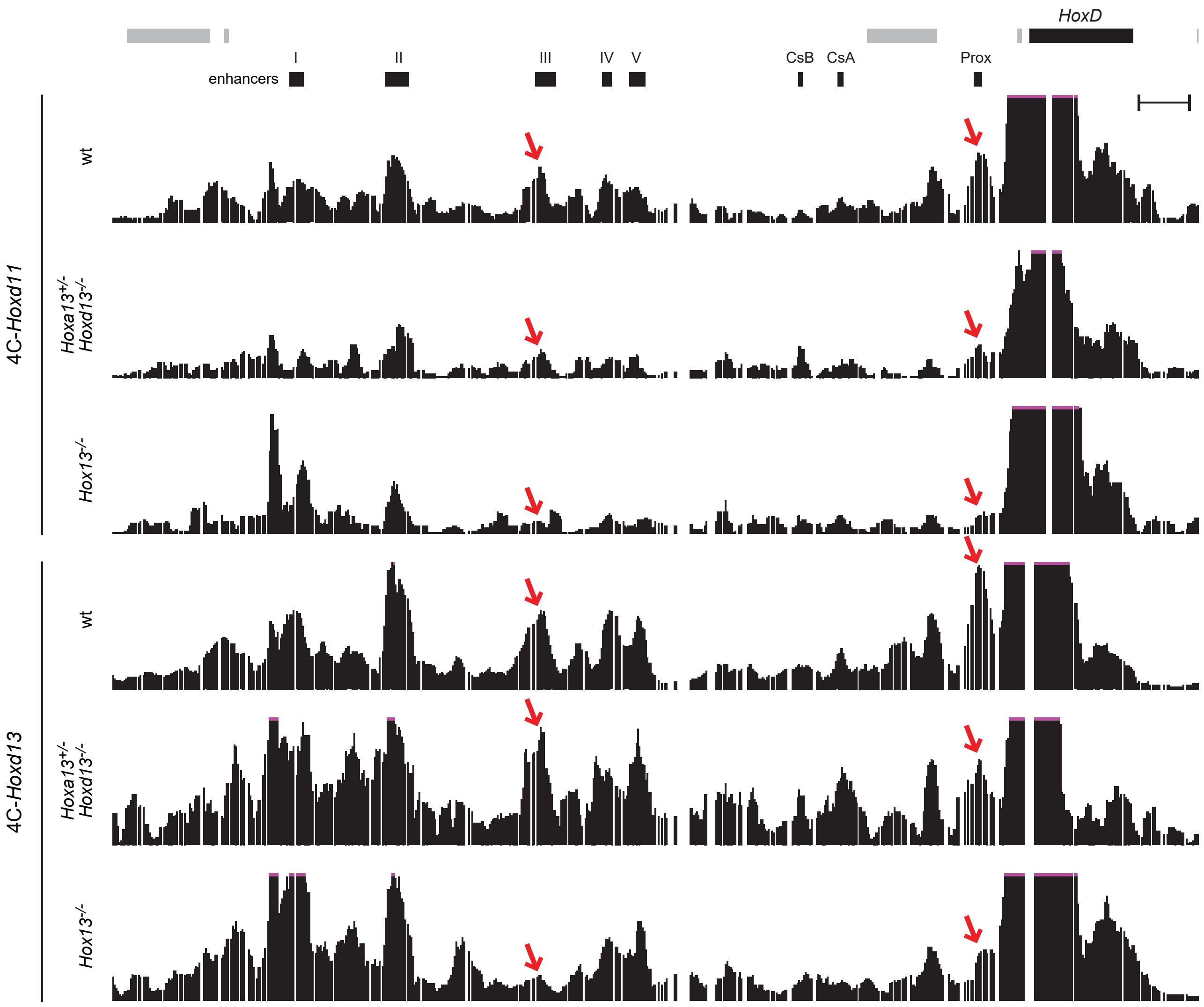

Figure S7. Lack of HOX13 function in distal limbs prevents contacts between *Hoxd11* or *Hoxd13* and digit-specific regulatory elements. The various tracks show the interaction profiles between either *Hoxd11* (tracks 1 to 3) or *Hoxd13* (tracks 4 to 6) and the centromeric regulatory landscape CDOM. Three genotypes are shown, including the wild type control, the double mutant condition and a heterozygous combination (indicated on the left). The *HoxD* cluster is shown as a blue rectangle (top) whereas grey boxes represent non-*Hox* coding genes. Below, previously identified regulatory islands located within C-DOM and controlling *Hoxd* gene expression in the distal limb bud domain are shown as black rectangles. The arrows points to two specific 4C contacts detected over the island III and Prox regulatory elements, previously described as hallmarks of C-DOM operating in developing digits (Lonfat et al., 2014). While these two interaction peaks are detected neither with *Hoxd11*, nor with *Hoxd13* as a bait, contacts are almost fully restored when a single copy of a *Hox13* gene is still active. The extent of the Y axis was adjusted proportionally to the maximum number of reads of the viewpoint in each sample. Scale bar: 50 kb.
